# Characteristics of Professionals Involved in Open Science in *Ecology and Evolution*

**DOI:** 10.1002/ece3.74595

**Published:** 2026-11-02

**Authors:** Zuzanna B. Zagrodzka, Thomas F. Johnson, Andrew P. Beckerman

**Affiliations:** ^1^ Ecology and Evolutionary Biology, School of Biosciences University of Sheffield Sheffield UK

## Abstract

Open science aims to improve research transparency, reuse and societal impact, yet its adoption and reach ultimately depend on people. Understanding how personal characteristics such as gender, work experience or professional role influence familiarity with, attitudes toward and engagement in open science is critical for the effective, widespread implementation of open science practices. We surveyed 342 individuals who produce, mobilise or use scientific knowledge in ecology and evolutionary biology. Their engagement was analysed using the open science change framework, which conceptualises open science as a progression from familiarity and perceptions to practices and support for societal goals, as outlined in the UNESCO Recommendations. Familiarity with open science emerged as a strongest and most consistent predictor of engagement: higher familiarity was associated with more positive attitudes, greater participation in open science practices and stronger agreement with UNESCO principles. Although these relationships are correlational and likely bidirectional, they highlight the central role of familiarity in open science engagement. Years of professional experience were associated with slightly less positive perceptions of open science, particularly among knowledge creators, while gender effects were weak across all stages of engagement. Stakeholder groups differed most in their familiarity with open science, while attitudes and support for broader goals were more similar once familiarity was considered. Together, these findings suggest that increasing familiarity with open science may be more effective than managing resistance or targeting specific demographic groups. Aligning incentives and practical support to increase familiarity offers actionable opportunities to strengthen open science engagement across the research ecosystem in ecology and evolutionary biology and enhance its societal impact.

## Graphical abstract

We surveyed 342 participants in ecology and evolutionary biology to examine how personal and professional characteristics relate to open science engagement. Familiarity with open science emerged as the strongest and most consistent predictor of positive attitudes, open science practices and support for UNESCO open science principles. Increasing familiarity may therefore represent an important target for interventions aimed at strengthening open science across the research ecosystem.

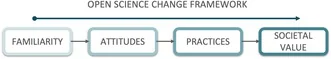

## 1 Introduction

Open science has been proposed as a key approach to improve knowledge mobilisation from knowledge creators (e.g., scientists) through to policy makers. Open science is fundamentally aligned with delivering evidence‐based policies (Olesk et al. 2019; Reichmann and Wieser 2022). Despite the central role of scientific evidence in addressing complex global challenges (National Research Council of the National Academies 2012), knowledge flow from knowledge generation to policy remains hindered by paywalls (International Association of Scientific, Technical, and Medical Publishers 2026), inaccessible or incomplete methods and data (Tedersoo et al. 2021; Page et al. 2022; Kambouris et al. 2024; Pethick et al. 2025), misalignment between research and policy needs (Oliver et al. 2014), slow research dissemination (O'Donnell et al. 2010; Nguyen et al. 2015; Christie et al. 2021) and conflicting stakeholders' interests (Zagrodzka et al. 2024). These persistent barriers show an urgent need for initiatives and interventions that improve the accessibility, transparency and usability of research.

Open science (also open research) defined by UNESCO as a set of principles and practices aimed at making scientific knowledge openly accessible, reusable and transparent—seeks to promote inclusivity, equity and democratisation of research (UNESCO 2021; Nosek et al. 2015; Cole et al. 2024). By strengthening research integrity and reproducibility, and by supporting fairer recognition of contributors and a more collaborative research culture (UNESCO 2021; Thibault et al. 2023; COARA 2025; Chtena et al. 2025) open science provides a foundation for more effective knowledge mobilisation.

However, the effectiveness of open science ultimately depends on the individuals involved in the creation, mobilisation, archiving, use or reuse of research outputs, regardless of their gender, employer (e.g., stakeholder in open science), level of seniority or job role. Understanding engagement with open science therefore requires not only examining institutional and structural conditions, but also the characteristics, experiences and positions of individuals who interpret, adopt and implement these practices in their work. Adoption of open science can be understood as a sequential process for individuals: individuals must first be familiar with open science concepts, then perceive them as beneficial, be able and motivated to integrate open practices into their workflows, and ultimately recognise their broader value for society (Cole et al. 2024) (see Figure 1). Although much attention has focused on researchers (Pardo Martínez and Poveda 2018; Soeharjono and Roche 2021; Ollé et al. 2023; Ferguson et al. 2023), far less is known about how individuals outside the research community perceive and engage with open science, despite their central role as users, interpreters and mobilisers of research.

**FIGURE 1:**
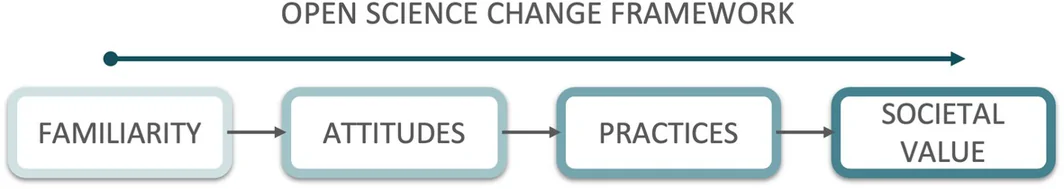
Open science change framework. This conceptual framework conceptualises open science as a progression from familiarity and perceptions to practices and support for societal goals. It was used to inform the structure of the survey.

Diversity among individuals within open science stakeholder communities (e.g., funders, knowledge brokers, infrastructure providers) may lead to substantial variation in awareness, expectations and uptake of open practices. Recent policies promoting or mandating open science suggest that familiarity might increase with career progression, but increased familiarity does not always translate into active involvement (Gownaris et al. 2022; Krauss et al. 2023). Established professionals may default to traditional workflows shaped by legacy incentive structures, such as researchers prioritising publication in high‐impact subscription‐based journals (Rowley et al. 2022) and thereby contribute to the oligopoly of academic publishers (Phelps 2022) or policy‐makers making decisions without direct engagement with scientific evidence (The Lancet Regional Health – Western Pacific 2022; Suazo‐Galdames et al. 2025) potentially reducing their motivation or perceived need to adopt open practices. Early‐career professionals, by contrast, may find it easier to incorporate new technologies, practices and workflows, place greater value on accessible research while still learning their profession (Berezko et al. 2021), and face stronger policy requirements to comply with open science expectations (Wróblewska et al. 2024). However, evidence also suggests that career precarity (Alderson et al. 2023), reliance on traditional metrics (Wróblewska et al. 2024; Alderson et al. 2023; Aubert Bonn and Pinxten 2021), structural constraints (Aubert Bonn and Pinxten 2021) and limited incentives (Roche et al. 2022; Mabile et al. 2025) can limit their capacity to prioritise and engage in open practices. These patterns highlight the importance of considering the characteristics of individuals within stakeholder communities both within and beyond academia.

Gender may also shape engagement with open science. Gender inequalities in organisations remain widespread (Piasna and Drahokoupil 2017; Farré et al. 2022; Dick 2024), influencing professional experiences and expectations. In science, women face systematic disadvantages that affect recognition and career progression (Cech 2022), including disparities in grant funding (van der Lee and Ellemers 2015; Witteman et al. 2019; Cruz‐Castro et al. 2023), publication acceptance (Salerno et al. 2019; Lerback et al. 2020), promotions (Moss‐Racusin et al. 2012; Régner et al. 2019), rewards (Mitchneck et al. 2016; Cruz‐Castro and Sanz‐Menendez 2021), authorship and credit attribution (Ni et al. 2021; Ross et al. 2022), as well as invisible emotional labour (Vial and Cowgill 2022). Women collaborate more (Bear and Woolley 2011; Abramo et al. 2013) and gender‐diverse teams also show higher creativity, innovation and collective intelligence (Bear and Woolley 2011; Riedl et al. 2021). Because women's communal orientation aligns with open science principles and their contributions are often undervalued or unrecognised, open science may be perceived as particularly rewarding, enhancing visibility, recognition and the likelihood of actively engaging in and promoting a positive research culture.

Building on the open science change framework (Figure 1), this study examines how engagement with open science varies among individuals involved in the ecological and evolutionary research ecosystem across multiple stakeholder/organisation/employer communities. Ecology and evolutionary biology are particularly relevant due to the data sharing practices, global collaboration and strong links to policy‐relevant challenges such as biodiversity loss and climate change. In addition, these fields have been at the forefront of adopting open research practices, supported by initiatives, communities and journal policies that promote transparency, reproducibility and data sharing. Together, these characteristics make ecology and evolutionary biology a valuable case study for examining open science engagement across diverse stakeholder groups. We conceptualise engagement with open science as a sequential process that progress from (1) familiarity with open science concepts, to (2) attitudes toward open science, to, if applicable, (3) participation in open science practices, and finally to (4) support for its broader societal benefits, as articulated in the UNESCO Recommendations on Open Science. The UNESCO Recommendation on Open Science was selected as the guiding framework for this study as it offers one of the most comprehensive and internationally endorsed conceptualisations of open science. Adopted by the UNESCO General Conference at its 41st session in 2021, it reflects broad international consensus on the principles, values and objectives underpinning open science. It also informs ongoing work on indicators and monitoring frameworks for assessing implementation, making it particularly well suited to empirical analyses of open science engagement. In this framework, each stage of engagement is analysed with years of experience as a key explanatory variable, familiarity with open science functioning both as an outcome of early engagement and as a predictor of subsequent stages, and gender and stakeholder group examined as potential effect modifiers. Consistent with this framework, we surveyed three groups: knowledge creators (research community), knowledge mobilisers in academia (e.g., publishers, journals, repositories, learned societies and funders) and knowledge mobilisers outside academia (e.g., government agencies, NGOs, industry and other brokers). We addressed the following research questions, each corresponding to a stage of the engagement framework:

1. Familiarity: Does the effect of years of experience on familiarity with open science vary by stakeholder group or gender?
2. Attitudes: Do years of experience and familiarity influence positive and negative associations with open science, and do these effects vary by stakeholder group or gender?
3. Practices: Does the effect of years of experience and familiarity on open science practices vary by gender or stakeholder group?
4. Societal benefits: Does the effect of years of experience and familiarity on agreement with UNESCO open science statements vary by gender or stakeholder group?

Understanding how demographic factors and professional roles shape engagement with open science is fundamental for designing targeted support, training and policy interventions. Such insights can help institutions, organisations and policymakers strengthen open science engagement across the research ecosystem and normalise open, collaborative knowledge mobilisation.

## 2 Materials and Methods

### 2.1 Survey

We created the survey using Qualtrics to assess respondents' familiarity with open science and the UNESCO Recommendations on Open Science (UNESCO 2021), their associations with open science, engagement in open science practices, and agreement with UNESCO statements on open science. We pre‐tested the survey with colleagues to ensure clarity and relevance of the questions.

The survey consisted of three sections. In the first part of the survey, we collected basic demographic information and stakeholder affiliations.

In the second section, we asked respondents to rate their familiarity with open science on a 0–10 scale, indicate their familiarity with the UNESCO Recommendations, and report engagement in open science practices (e.g., Open Access, FAIR data, Open Licences). We also asked respondents to indicate whether they associated a set of positive (e.g., collaboration, efficiency, trust) and negative (e.g., frustration, time cost, risk of scooping) terms with open science, based on prior literature (Soeharjono and Roche 2021; Arza and Fressoli 2017; Abele‐Brehm et al. 2019).

In the third section, we asked respondents to rate their agreement with 11 statements adapted from the UNESCO Recommendation on Open Science on a 0–10 scale (Zagrodzka 2024). Table 1 summarises these statements and their descriptive labels.

**TABLE 1:** Survey statements on open science and their descriptive labels, adapted from the UNESCO Recommendation on Open Science.

Sentence	Description
Access to scientific knowledge should be as open as possible.	Open access
Scientific outputs (e.g., publications, research data and software, educational resources) should be available in the public domain under an open licence.	Open licencing
Greater access to scientific processes and outputs increase the social impact of science.	Social impact
Open science could improve the evidence needed for robust decision‐making and policy.	Evidence‐based policy
Open science could increase trust in science.	Increasing trust in science
Open science contributes to democratisation of knowledge.	Knowledge democratisation
Open science services should be viewed as essential research infrastructures, governed and owned by the community.	Essential OS services
Open science services should be funded collectively by governments, funders and institutions.	Collective OS funding
Open science contributes to fighting misinformation and disinformation.	Fighting misinformation
Open science contributes to addressing existing systemic inequalities and enclosures of wealth, knowledge and power.	Addressing systemic inequalities
Open science contributes to guiding scientific work toward solving problems of social importance.	Addressing social challenges

Participation in the survey was voluntary, and respondents were informed that they could withdraw at any time without providing a reason. The University of Sheffield Ethics Review Procedure approved the study (Application 058550).

### 2.2 Recruitment and Participants

We identified organisations and individuals working in, supporting, funding, publishing or using ecology and evolutionary biology research and data. We sent the survey directly to approximately 6700 successfully delivered email addresses (excluding bounced emails), including individuals and organisational contacts, where publicly available contact information was identified through multiple sources (e.g., authors of ecology and evolutionary biology publications (2023), professional networks, community groups and mailing lists). While recruitment focused primarily on stakeholders involved in ecology and evolutionary biology, we also contacted individuals from broader open research, research improvement and research support communities where the study was considered relevant and could be shared further through professional networks. We also shared the survey publicly via X, LinkedIn, Slack communities (e.g., SORTEE, Evolution & Ecology Seminars), relevant forums and email lists, and encouraged snowballing to increase participation. Consequently, our participant group is diverse but not statistically representative of a clearly bounded population, with numerous responses from the Universities and Research Institutions and comparatively fewer responses from other stakeholder groups (see Section 4.6 for details). The survey was open from April 23rd to August 12th, 2024, and closed after a sustained period with no new responses. A total of 342 participants completed the survey, representing a variety of stakeholder groups. Respondents indicated their primary affiliation from nine categories: Research Institutes, Research Funders, Publishers, Journals, Learned Societies, Repositories, Governments, Commercial/Industry and ‘Other’ (e.g., charities). To address the low participation rate outside of universities and research institutes, we grouped stakeholders based on their role in the knowledge cycle and landscape, which enabled statistical analyses. The groups include: knowledge creators (primarily affiliated with the Universities and Research Institutes), knowledge mobilisers in academia (Research Funders, Publishers, Journals, Learned Societies and Repositories) and knowledge mobilisers outside academia (Governments, Commercial/Industry and Other).

We collected demographic information including gender, career stage, years of experience, and primary and secondary stakeholder affiliations. We also recorded respondents' geographic location (Figure 2A), though we did not include geography in subsequent models.

**FIGURE 2:**
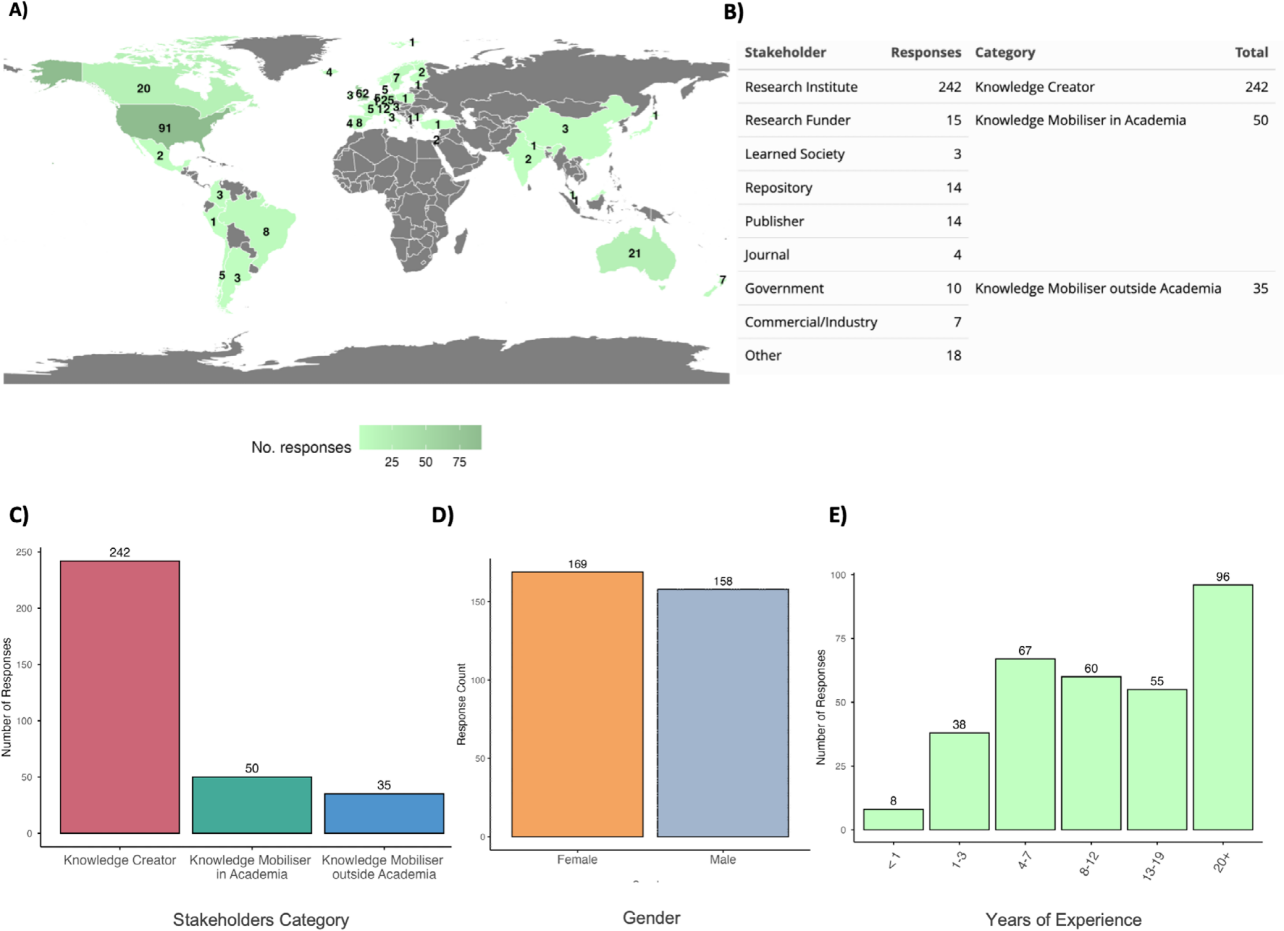
Demographic information of survey respondents. (A) Geographic distribution of respondents. The map indicates the countries in which survey respondents work. (B) Primary affiliation of respondents. The table shows the number of responses by primary stakeholder affiliation and by newly assigned stakeholder categories. (C) Stakeholder categories. Plot showing the number of responses within each newly assigned category. (D) Gender of respondents. (E) Years of professional experience. Distribution of respondents according to years of professional experience.

### 2.3 Predictions

We developed four main hypotheses. First, we expected that self‐reported familiarity with open science would increase with years of professional experience, as more experienced individuals are more likely to encounter open science practices and related policies. Second, we hypothesised that women would exhibit higher familiarity, more positive associations, and greater engagement in open science practices than men, potentially influenced by experience. Third, we anticipated differences across stakeholder categories, with knowledge creators showing higher familiarity but potentially more nuanced or critical perspectives due to practical experience. Finally, we predicted that respondents with increased years of experience and therefore greater familiarity with open science would hold more positive attitudes and report higher agreement with the UNESCO Recommendations.

We included interaction terms in our models to examine whether the effect of years of experience varied by gender and stakeholder group. We did not have strong a priori hypotheses regarding the direction of effects.

### 2.4 Data Analysis

We conducted all analyses in R (version 4.4.2) using the brms package (version 2.22.0) (Bürkner 2017, 2018, 2021) for Bayesian regression modelling. Bayesian models allowed us to estimate relationships between predictors and response variables while explicitly quantifying uncertainty in parameter estimates (see Zagrodzka 2026 for code).

We first excluded incomplete survey responses and participants who reported not working in ecology or evolutionary biology. Three respondents did not provide information on their years of professional experience and were excluded from the models, as this variable was included in all analyses. No missing values were imputed. We also removed categories with very small sample sizes that would not support reliable parameter estimation. Our original intention was to model years of experience as an ordered categorical predictor. However, given our modest sample size, especially outside of the academic stakeholder group, this was no longer viable as it would require estimating a separate parameter for each category. To avoid over‐parameterisation and retain ordinal information, we converted years of experience to a numeric variable by assigning each category its midpoint, with the open‐ended categories (‘< 1 year’ and ‘> 20 years’) coded as 0 and 20, respectively. For beta regression models (Models 1 and 5), we rescaled Likert‐scale responses from 0 to 10 to the unit interval, adjusting values of 0 and 1 by ±0.001 to meet the distributional requirements of the beta distribution. We used Bernoulli regression for binary outcomes (Models 2–4).

Model 1 examined factors associated with familiarity with open science. The response variable was familiarity with open science, and the predictors were gender, stakeholder group and years of experience. Models 2 and 3 examined factors associated with positive and negative perceptions of open science, respectively. The response variables were positive perceptions (Model 2) and negative perceptions (Model 3), while the predictors were gender, stakeholder group, years of experience and familiarity with open science. Model 4 examined factors associated with engagement in open science practices. The response variable was open science practices, and the predictors were gender, years of experience and familiarity with open science. This model included only knowledge creators. Model 5 examined factors associated with agreement with the UNESCO statements on open science. The response variable was agreement with the UNESCO statements, and the predictors were gender, stakeholder group, years of experience and familiarity with open science. Random effects were included in Models 2–5 to account for repeated responses from the same participants. Weakly informative Bayesian priors were used throughout. Full details of the model structures, priors, response distributions and predictors are provided in Table 2.

**TABLE 2:** Summary of model specifications. Categorical predictors included stakeholder group (S) and gender (G), while continuous predictors included years of experience (Y) and familiarity with open science (F).

Question	Model	Response variable	Type	Predictors	Interaction terms	Random effects	Family and link
Q1	M1	Familiarity	Continuous	G, S, Y	G:Y, S:Y	—	Beta, logit
Q2	M2	Positive perceptions	Binary	G, S, Y, F	G:Y, S:Y	Random ID, Term (positive)	Bernoulli, logit
Q3	M3	Negative perceptions	Binary	G, S, Y, F	G:Y, S:Y	Random ID, Term (negative)	Bernoulli, logit
Q4	M4	Practices	Binary	G, Y, F	G:Y	Random ID, Practice	Bernoulli, logit
Q4	M5	UNESCO sentences	Continuous	G, S, Y, F	G:Y, S:Y	Random ID, Sentence	Beta, logit

We ran four Markov chain Monte Carlo (MCMC) chains with 6000 iterations each, including 2000 warm‐up iterations, yielding 16,000 post‐warm‐up samples. We performed parallel computation across four CPU cores and set a fixed random seed (1234) to ensure reproducibility. We version‐controlled all analysis scripts on GitHub and archived the final repository on Zenodo (Zagrodzka 2026). Table 2 summarises the full model specifications, and response variables correspond to the survey items.

## 3 Results

### 3.1 Respondent Characteristics

We received 342 completed responses, representing all three aggregated stakeholder categories: knowledge creators (*n* = 242), knowledge mobilisers within academia (*n* = 51) and knowledge mobilisers outside academia (*n* = 37, Figure 2B,C). Respondents were nearly evenly split by gender, with 49.4% identifying as female (*n* = 169) and 46.2% as male (*n* = 158, Figure 2D). Non‐binary respondents (*n* = 9) and those who did not disclose gender (*n* = 6) were excluded from analyses due to small sample sizes. Professional experience varied, with the largest group reporting more than 20 years of experience (29.5%, *n* = 100). Other ranges included 4–7 years (20.6%, *n* = 70), 8–12 years (18.3%, *n* = 62), 13–19 years (17.1%, *n* = 58), 1–3 years (11.8%, *n* = 40) and less than 1 year (2.65%, *n* = 9, Figure 2E).

We plotted respondents' geographic distribution (Figure 2A) to demonstrate relatively diverse representation across multiple continents, though geographic location was not included in subsequent models.

### 3.2 Familiarity With Open Science and UNESCO Recommendations

We found that self‐reported familiarity with open science (0–10 scale; *N* = 327) was moderate to high, with 72% of respondents scoring between 6 and 10. The median score was 8 (IQR = 6–9). Awareness of the UNESCO Recommendations varied, with 39 respondents (11.9%) reporting high familiarity, 134 respondents (41.0%) indicating awareness and 154 respondents (47.1%) reporting no familiarity.

### 3.3 Familiarity With Open Science

#### 3.3.1 Does the Effect of the Years of Experience on Familiarity on Open Science Vary by Stakeholder Group or Gender?

Familiarity with open science increased with years of professional experience (Model 1; Figure 3A–D; Table S1.1). The main effect of years of experience on familiarity was positive and credibly different from zero (posterior mean *β* = 0.03, 95% credible interval [0.002, 0.059]). However, the effect size was small, indicating only modest increases in familiarity across professional experience. This relationship was consistent across genders and stakeholder groups, as interaction terms between experience and gender and between experience and stakeholder role were small and with credible intervals spanning zero.

**FIGURE 3:**
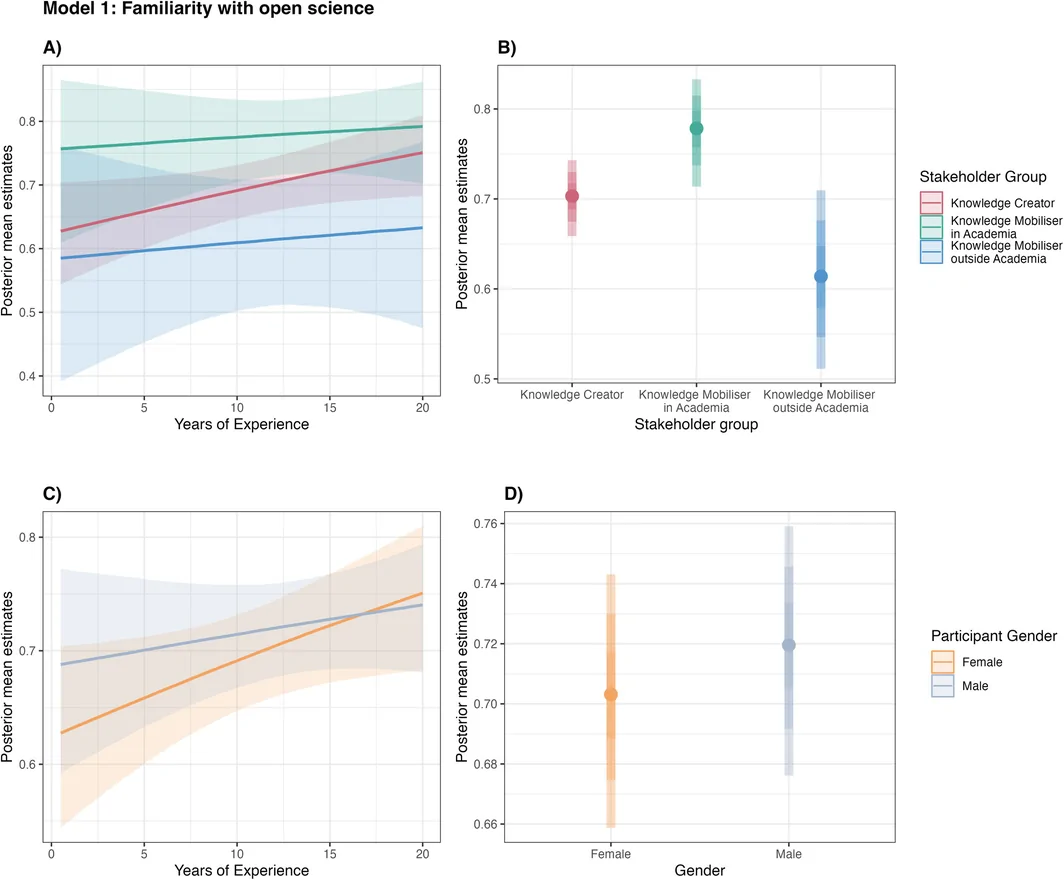
Posterior mean estimates from Model 1 fitted to survey data. Panels display posterior mean predictions and group‐level estimates with associated uncertainty. Across all panels, solid lines and points represent posterior mean estimates. Predictions across years of professional experience are shown as posterior means with shaded 95% credible intervals, while group‐level averages are shown as posterior medians with error bars indicating 50%, 80% and 95% credible intervals.

Overall familiarity with open science was high across respondents. Knowledge mobilisers outside academia reported lower average familiarity than knowledge creators (posterior mean *β* = −0.362, 95% CI [−0.806, 0.089]), whereas familiarity among knowledge mobilisers within academia did not differ credibly from that of knowledge creators (Figure 3A,B). Full model estimates and credible intervals are provided in Supplementary Information S1 (Model 1).

### 3.4 Positive and Negative Perceptions of Open Science Terms

#### 3.4.1 Does the Effect of Years of Experience, Familiarity With Open Science Influence Positive and Negative Perceptions of Open Science Terms and Varies by Stakeholder Group or Gender?

Familiarity with open science was a strong predictor of positive perceptions (Model 2; *β* = 1.179, 95% CI [0.392, 1.964], Table S1.2), but was not credibly associated with negative perceptions in the corresponding model (Model 3; *β* = −0.229, 95% CI [−0.984, 0.519], Table S1.3).

In contrast, years of professional experience were associated with less favourable perceptions overall, reflected in both positive perceptions declining with increasing experience, that is, less positive associations with aspects of open science (Model 2; posterior mean *β* = −0.054, 95% CI [−0.094, −0.013], Table S1.2), and negative perceptions increasing with experience, that is, respondents experience or associate more negative aspects of open science (Model 3; posterior mean *β* = 0.056, 95% CI [0.018, 0.095], Table S1.3). These effects are marginal and estimated assuming familiarity remained unchanged across survey participants. This is important as familiarity is positively associated with both years of experience and positive perceptions, and so the negative effect of years of experience on perceptions is only marginal. In practice, the more experienced you are, the more likely you are to be familiar with open science, and more likely you are to have favourable perceptions, that is, the total effect of experience on perceptions may be positive given its role on familiarity.

There was limited evidence for moderation by stakeholder groups, although two interaction effects were credibly different from zero. First, we found that positive perceptions (Model 2; Figure 4A–D) increased more strongly with experience among knowledge mobilisers outside academia compared with knowledge creators (*β* = 0.090, 95% CI [0.007, 0.173]). This suggests that years of experience may shape certain aspects of open science that are perceived as positive among knowledge mobilisers outside academia. In particular, some positive and negative perceptions that are commonly associated with open science by knowledge creators (e.g., time and financial costs) may be understood, experienced, or associated differently by individuals working outside academic settings, reflecting differences in roles and forms of involvement rather than differences in overall favourability. Second, negative perceptions (Model 3; Figure 5A–D) increased less steeply with experience among knowledge mobilisers within academia (*β* = −0.114, 95% CI [−0.189, −0.038]) relative to knowledge creators. Although statistically credible, both interaction effects were small in magnitude, and all other stakeholder by years of experience interactions were small and uncertain.

**FIGURE 4:**
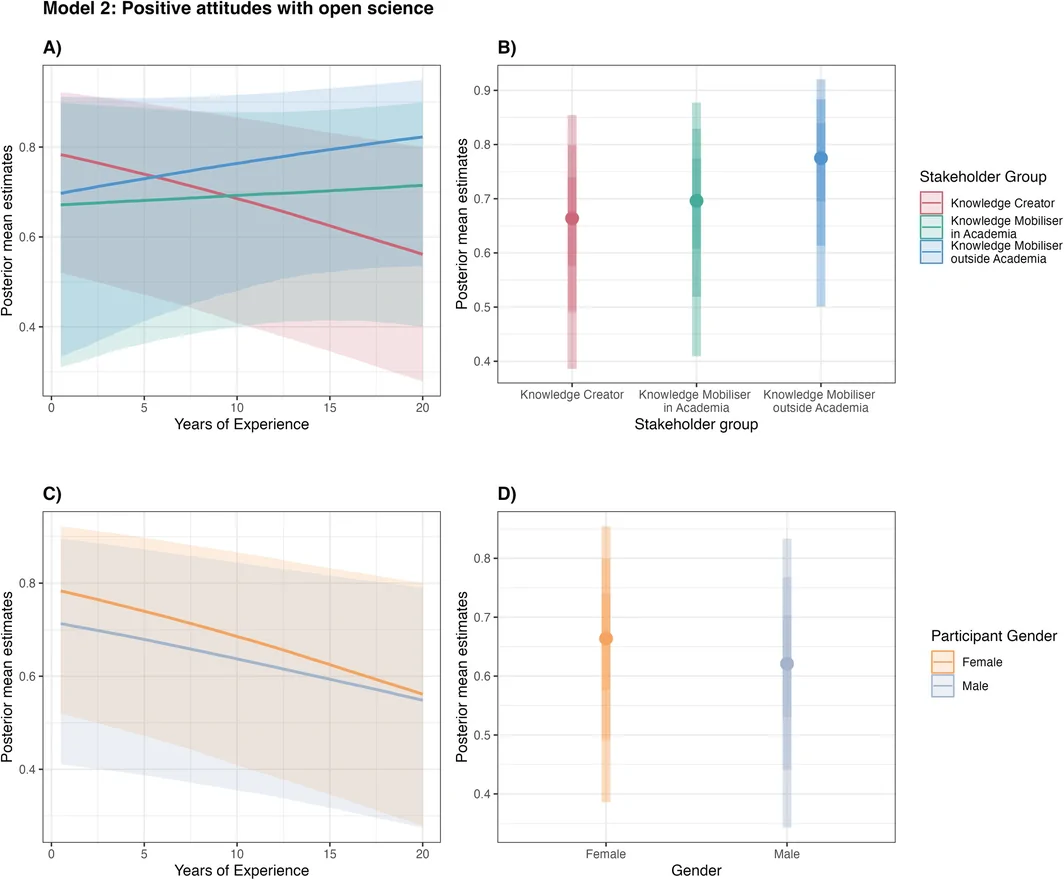
Posterior mean estimates from Model 2 fitted to survey data. Panels display posterior mean predictions and group‐level estimates with associated uncertainty. Across all panels, solid lines and points represent posterior mean estimates. Predictions across years of professional experience are shown as posterior means with shaded 95% credible intervals, while group‐level averages are shown as posterior medians with error bars indicating 50%, 80% and 95% credible intervals.

**FIGURE 5:**
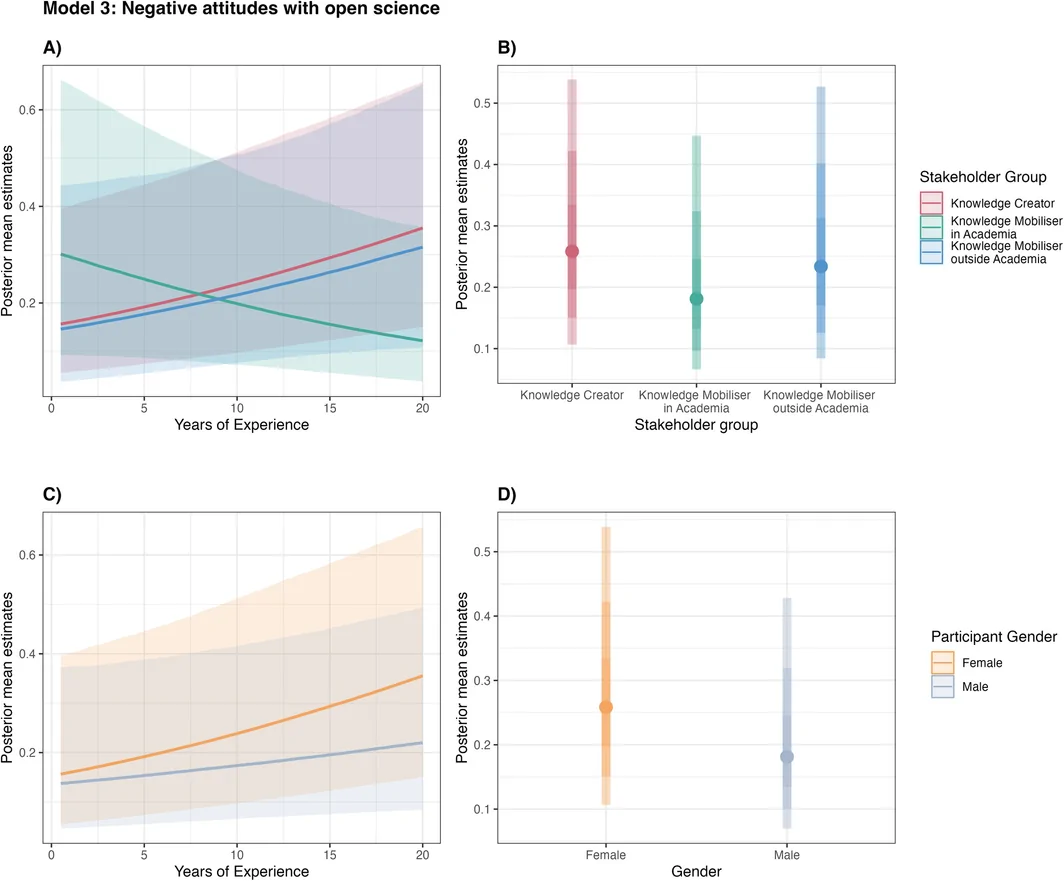
Posterior mean estimates from Model 3 fitted to survey data. Panels display posterior mean predictions and group‐level estimates with associated uncertainty. Across all panels, solid lines and points represent posterior mean estimates. Predictions across years of professional experience are shown as posterior means with shaded 95% credible intervals, while group‐level averages are shown as posterior medians with error bars indicating 50%, 80% and 95% credible intervals.

Additionally, gender differences were evident for negative perceptions, with males reporting higher negative perceptions than women, though the estimated effect was very weak (Figures 4C,D and 5C,D).

Respondents most frequently associated open science with positive attributes such as increased visibility, trust, collaboration and efficiency, while concerns about financial and time costs were also common (Figure 6). Few respondents associated open science with negative terms such as misuse or scooping.

**FIGURE 6:**
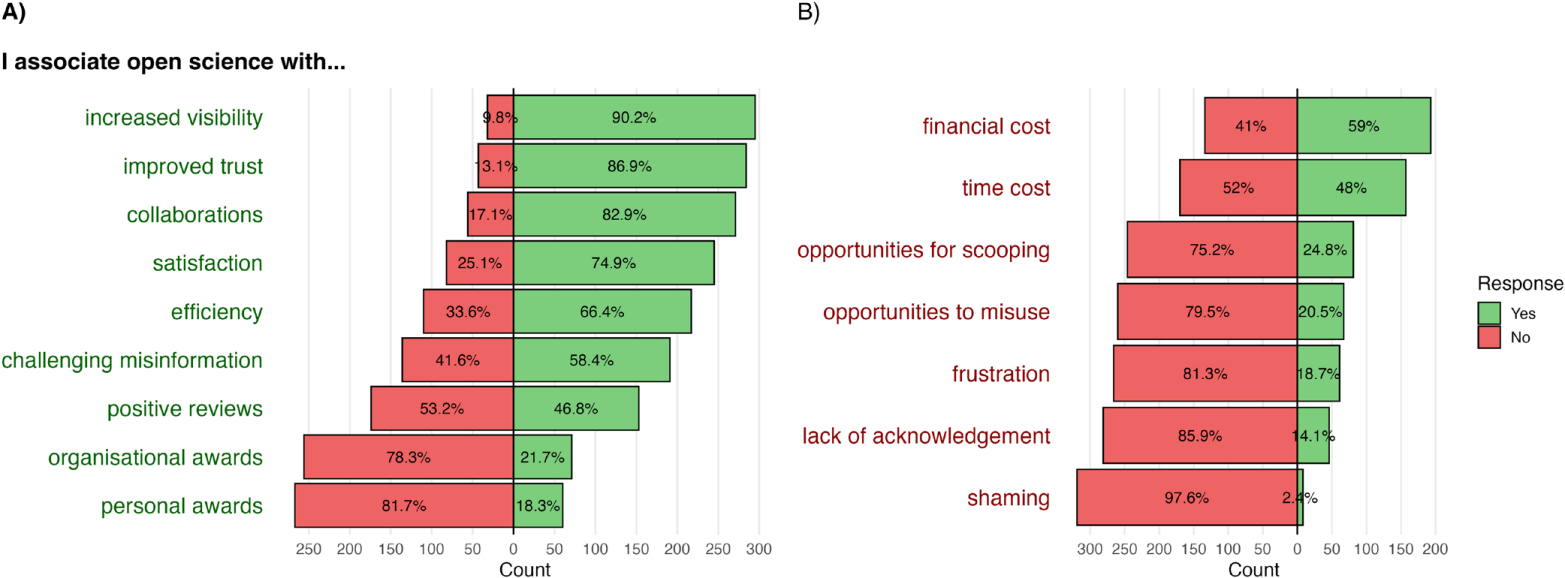
Positive and negative associations with open science. Proportion of respondents associating predefined positive and negative terms with open science. Terms are grouped by conceptual categories: Positive on the left and negative associations on the right.

### 3.5 Engagement With Open Science Practices

#### 3.5.1 Does the Effect of Years of Experience and Familiarity on Open Science Practices Vary by Gender?

Among respondents affiliated with universities and research institutes, engagement was highest for open access (51.8%) and FAIR data (47.7%), followed by open methods (28.2%), with lower uptake of open education (15.3%) and CARE principles (11.0%) (Figure 7). A small yet comparatively high proportion of respondents (8.8%) reported no engagement with any of the listed practices.

**FIGURE 7:**
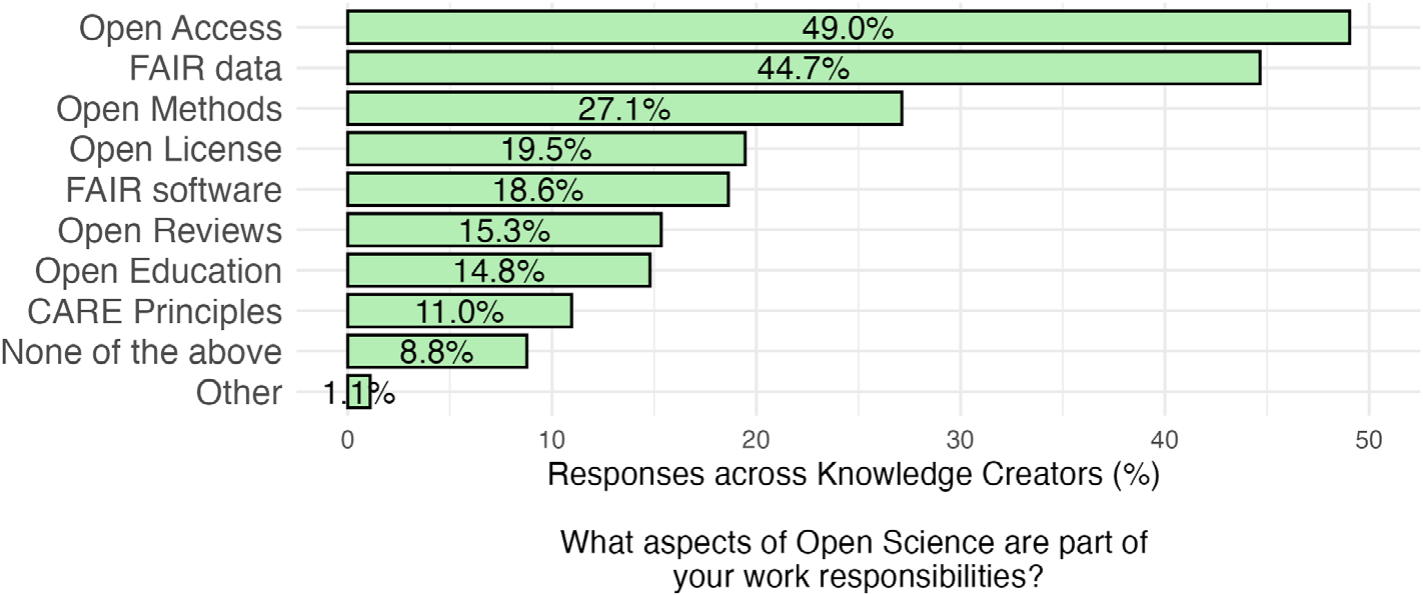
Engagement in open science practices. Percentage of respondents affiliated with universities and research institutes reporting engagement in specific Open Science practices as part of their work responsibilities, based on responses to the question: ‘Which aspects of Open Science are part of your work responsibilities?’

Engagement in open science practices was positively associated with familiarity with open science (Model 4; Table S1.4). Familiarity showed a credible positive association with engagement (posterior mean *β* = 0.158, 95% CI [0.049, 0.267], log‐odds scale). In contrast, the main effect of years of professional experience was small and uncertain (*β* = 0.014, 95% CI [−0.034, 0.062]). While the estimated association with familiarity was modest, it was considerably stronger than the corresponding effect of years of professional experience, which was small and uncertain. As expected from the cross‐sectional design of the survey, these analyses quantify associations rather than the direction of influence between familiarity and engagement.

There was little evidence that engagement differed substantially by gender. Estimated baseline engagement was similar for male (*β* = −1.654, 95% CI [−2.881, −0.403]) and female respondents (*β* = −1.814, 95% CI [−3.002, −0.593]). The interaction between gender and years of experience was weak and uncertain (*β* = −0.053, 95% CI [−0.122, 0.016]; Figure 8A,B).

**FIGURE 8:**
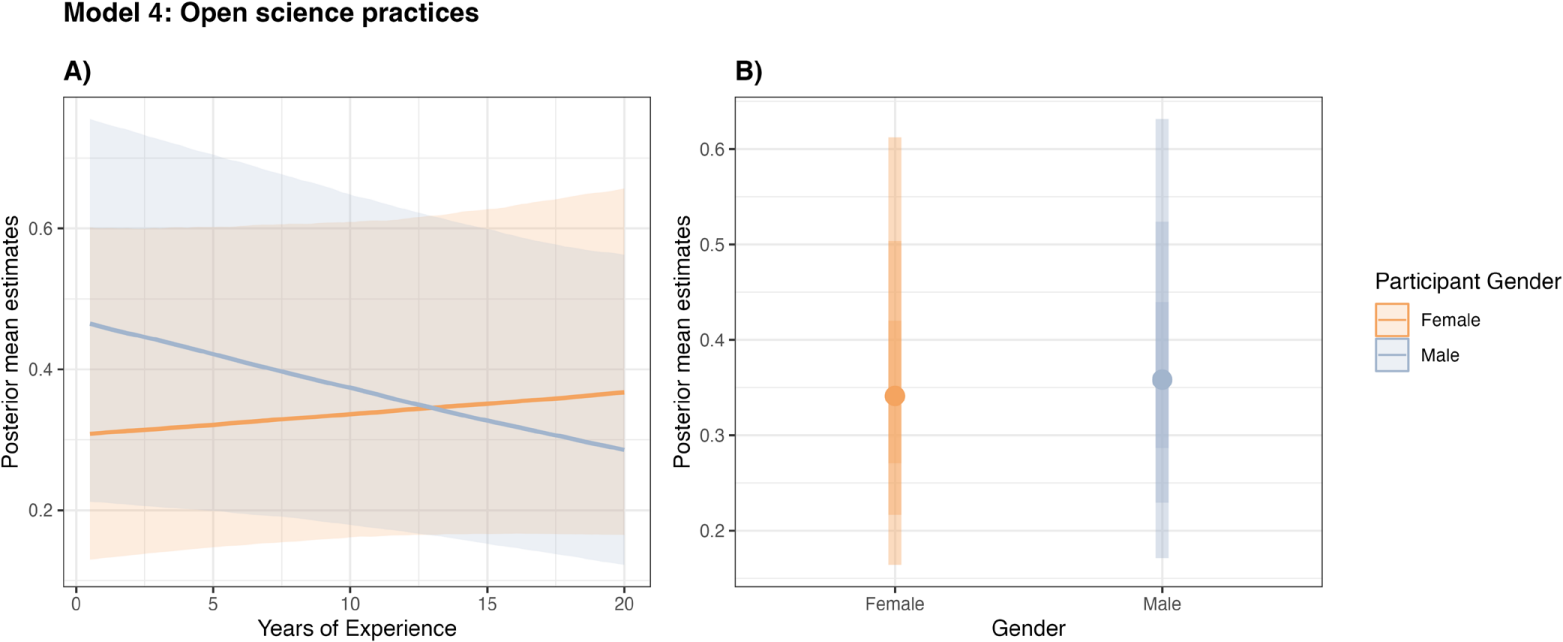
Posterior mean estimates from Model 4 fitted to survey data. Panels display posterior mean predictions and group‐level estimates with associated uncertainty. Across all panels, solid lines and points represent posterior mean estimates. Predictions across years of professional experience are shown as posterior means with shaded 95% credible intervals, while group‐level averages are shown as posterior medians with error bars indicating 50%, 80% and 95% credible intervals.

### 3.6 Agreement With UNESCO Open Science Statements

#### 3.6.1 Does the Effect of Years of Experience, Gender and Familiarity on Open Science on Agreement With the UNESCO Statements Vary by Stakeholder Group?

Most respondents expressed agreement with statements derived from the UNESCO Recommendations on Open Science (UNESCO 2021), particularly those emphasising open access to scientific knowledge (Figure 9). Agreement was slightly lower for statements related to addressing societal challenges and combating misinformation, although more than 70% of respondents still expressed agreement.

**FIGURE 9:**
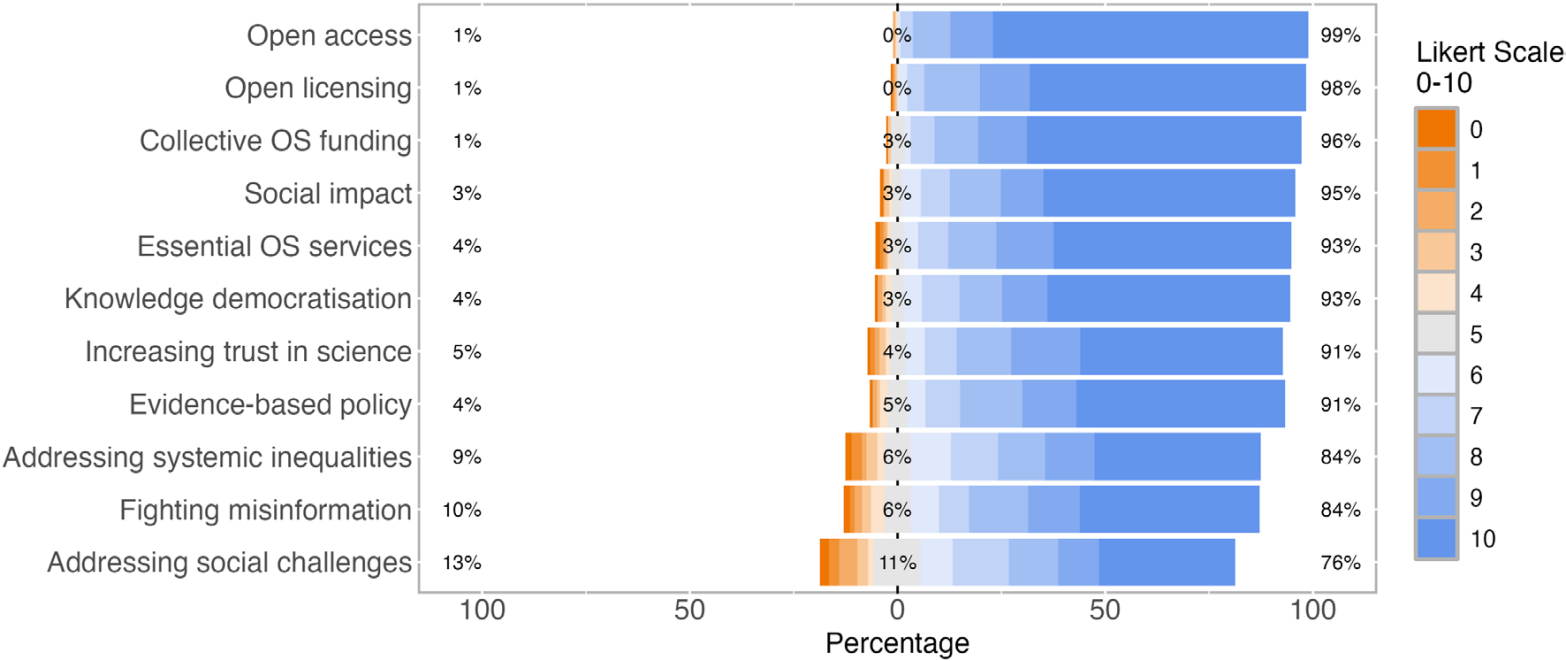
Agreement with UNESCO open science statements. Percentage of responses on a 0–10 Likert scale (0—strongly disagree, 10—strongly agree).

Beta regression models indicated that familiarity with open science was strongly and positively associated with agreement with UNESCO open science statements (Model 5; Table S1.5). Familiarity exhibited a robust positive effect (posterior mean *β* = 0.756, 95% CI [0.325, 1.181]). Years of professional experience, by contrast, showed a small but negative association with agreement (*β* = −0.031, 95% CI [−0.052, −0.010]).

Stakeholder groups' differences were pronounced, with all groups showing higher agreement relative to the reference category, particularly knowledge mobilisers outside academia (*β* = 1.764, 95% CI [1.283, 2.253]). Gender effects were small and uncertain (*β* = −0.140, 95% CI [−0.337, 0.055]). Interaction terms between experience and gender or stakeholder group were likewise weak and uncertain, with credible intervals spanning zero (Figure 10A–D).

**FIGURE 10:**
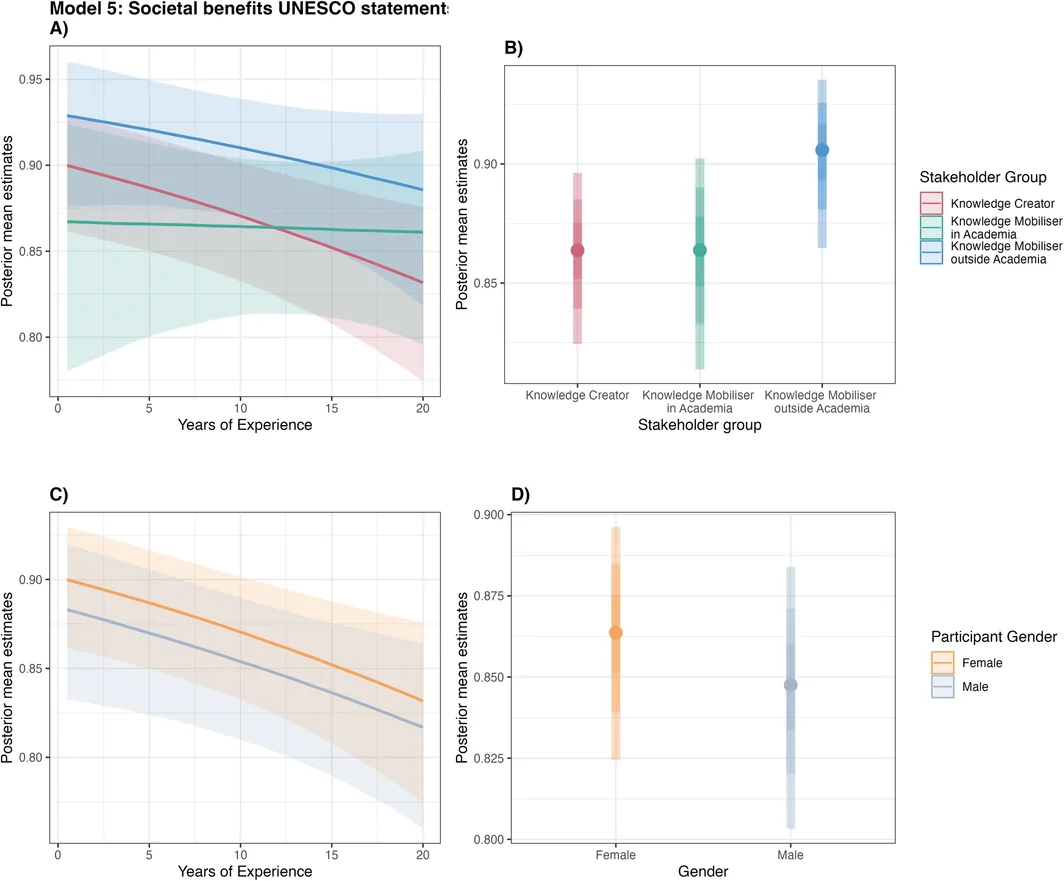
Posterior mean estimates from Model 5 fitted to survey data. Panels display posterior mean predictions and group‐level estimates with associated uncertainty. Across all panels, solid lines and points represent posterior mean estimates. Predictions across years of professional experience are shown as posterior means with shaded 95% credible intervals, while group‐level averages are shown as posterior medians with error bars indicating 50%, 80% and 95% credible intervals.

## 4 Discussion

Open science is widely promoted as a way to improve transparency, reliability and reuse of research, while also enabling broader societal uptake of scientific knowledge (UNESCO 2021; Nosek et al. 2015). Understanding who engages with open science, and why, is therefore essential for designing effective policies and support mechanisms. By examining familiarity, attitudes, practices and alignment with open science goals across gender, career stage and stakeholder groups, this study provides insight into the characteristics of people involved in engagement with open science in ecology and evolutionary biology. Across the first three stages of engagement (1) familiarity, (2) attitudes, and (3) practices, familiarity with open science itself emerged as the most consistent factor associated with engagement. Familiarity was first shaped only weakly by years of professional experience and showed little variation by gender, but it was then strongly associated with both attitudes toward open science and participation in open science practices. Differences between stakeholder groups were most evident at the level of familiarity with open science, but were weaker for attitudes and practices once familiarity was taken into account. In contrast, agreement with the broader societal goals articulated in the UNESCO Recommendations on Open Science was strongly associated with familiarity with open science, and increased with years of professional experience, with little variation by gender or stakeholder group. Although these associations are correlational and likely bidirectional, they consistently point at familiarity as a central correlate of open science engagement, regardless of whether familiarity drives engagement or engagement increases familiarity. Together, these findings suggest that differences in professional roles and work environments shape how individuals come across and engage with open science, but that support for its broader societal value is widespread and closely associated with familiarity and understanding of open science principles.

### 4.1 Familiarity With Open Science (RQ1)

Self‐reported familiarity with open science was generally high across respondents, suggesting that open science is no longer a niche or emerging concept within ecology and evolutionary research. Familiarity showed only a weak association with years of professional experience and did not differ meaningfully by gender. This indicates that awareness of open science has been spreading broadly across career stages, likely reflecting the growing visibility of open science requirements in funder policies and journal guidelines over the past decade, with expectations around open access publishing, data sharing and transparency across the research lifecycle increasingly reinforced through international, regional and national policy (UNESCO 2021, 2022; SPARC 2026; European Commission 2026).

Differences across stakeholder groups were more pronounced. Knowledge mobilisers outside academia reported lower familiarity than academic stakeholders, despite expressing comparable levels of support for open science principles. This suggests that while the normative goals of open science resonate beyond academia, its terminology, infrastructures and formal practices (e.g., transparent and unbiased methodologies, available data) remain largely embedded within academic contexts. Given the growing emphasis on evidence‐informed policymaking and science‐policy interfaces, particularly in areas such as health and climate change, this gap in familiarity may limit the effective uptake and reuse of research outside academia (Fears et al. 2021; Hadorn et al. 2022). Improving transparency and robustness in ways that are legible and usable beyond academia could therefore maximise the societal impact of open science.

### 4.2 Attitudes Toward Open Science and Perceived Costs (RQ2)

Respondents generally associated open science with positive outcomes, including increased trust, visibility and collaboration. Concerns commonly raised in the research community, such as scooping or misuse, were not prominent in this study. Instead, the most perceived barriers were time and financial costs, and the absence of personal or institutional rewards.

Among knowledge creators, positive associations tended to decrease slightly with increasing years of experience (when familiarity on open science was unchanged), while negative associations increased modestly. This pattern is consistent with prior research showing that senior researchers often operate within entrenched incentive structures (Rawat and Meena 2014; Link 2015; Farrow 2025), where practices emphasising productivity metrics and publication volume may conflict with the additional labour required for open science (Farrow 2025). This does not indicate opposition to openness, but rather highlights a structural misalignment between open science expectations and prevailing evaluation systems.

Despite expectations that gender differences might emerge, attitudes toward open science did not vary strongly by gender, although men reported marginally higher negative perceptions. This is notable in light of extensive evidence that, compared to men, women's contributions in science are less likely to be recognised or credited (Ross et al. 2022), particularly for collaborative (Bear and Woolley 2011; Abramo et al. 2013) and technical work (Macaluso et al. 2016) that aligns closely with open science practices but remains undervalued in current reward structures. Additionally, women face other systemic barriers in academic careers, including a higher likelihood of leaving STEM fields before reaching senior positions (Holman et al. 2018). Greater recognition and institutional support for open and collaborative research practices may therefore have the potential to contribute not only to more equitable credit allocation but also to improved retention of women in academia as they progress toward senior roles. One possible explanation for the weak gender differences observed here is that, while open science has been proposed as a mechanism for recognising a broader range of research contributions and supporting more equitable research cultures, these benefits may not yet be clearly perceived or realised in practice to influence attitudes or engagement (see the next section). If the potential career progression, increased recognition, and cultural benefits associated with open science are not yet clearly experienced by researchers, they may not provide a strong incentive for differential engagement across genders.

### 4.3 Engagement in Open Science Practices (RQ3)

Engagement in open science practices across the knowledge creators was uneven. Open access publishing and FAIR data practices were relatively common, likely reflecting funder and journal mandates that increasingly require compliance (Huang et al. 2020; Ivimey‐Cook et al. 2025). In contrast, practices such as open reviews, open licence or methods were less frequently reported. A small proportion of respondents reported no engagement with any listed practices, indicating that despite policy changes, some members of the research community do not engage with open science practices.

Familiarity with open science was the strongest predictor of engagement, showing the strongest positive association with participation in open science practices. Although these associations are likely bidirectional, they suggest that familiarity, knowledge, capacity and support are closely linked to participation. It is equally plausible that engaging in open science practices increases familiarity with open science concepts and terminology, reinforcing the close relationship between the two. Gender did not significantly structure engagement. Taken together, these findings suggest that increasing familiarity and reducing practical barriers may be more effective than targeting specific demographic groups. They also align with evidence that earlier career stages are more formative for adopting new research norms, whereas established researchers may find it more difficult to shift practices shaped by long‐standing metric‐driven incentives (Rawat and Meena 2014; Link 2015; Farrow 2025).

### 4.4 Support for UNESCO Open Science Goals (RQ4)

Most respondents agreed with the statements from the UNESCO Recommendations on Open Science (UNESCO 2021), particularly regarding open access to scientific knowledge. Agreement was somewhat lower for statements relating to addressing societal challenges and combating misinformation, although overall support remained high. Familiarity with open science and years of professional experience were positively associated with agreement, while gender and stakeholder groups had limited influence.

This pattern is consistent with research highlighting the potential of open and transparent research to strengthen trust in science, improve research quality and tackle misinformation, particularly in policy‐relevant and crisis contexts (Fears et al. 2021; Hadorn et al. 2022; Hopf et al. 2019). Open practices can enhance accountability, reproducibility and credibility, all of which are critical in addressing declining trust and the spread of false or misleading scientific claims.

At the same time, awareness of the UNESCO Recommendations on Open Science document (UNESCO 2021) remained relatively low. Five years after their adoption, this gap between governmental commitment and practitioner awareness highlights the need for clearer communication about the societal rationale for open science and the role of the researcher community within this broader policy landscape.

### 4.5 From Findings to Action: Recommendations

In this section, we reflect on findings from our study to identify potential priorities for strengthening open science engagement across the research ecosystem and to promote open science more effectively.

First, familiarity matters. Increased familiarity was consistently associated with more positive attitudes, greater engagement in practices, and stronger support for open science principles. Although our study cannot determine the direction of these associations, familiarity is a central correlate of engagement and may represent a practical and potentially modifiable point for interventions aimed at strengthening engagement. Increased familiarity may support engagement, but engagement itself may also increase familiarity. Targeted training and guidance for researchers transitioning into new career stages and raising awareness outside academia could therefore have cascading benefits.

Second, incentives need to be aligned with effort. The lack of perceived personal or institutional rewards, combined with concerns about time and financial costs, suggests that open science is still often experienced as unpaid labour. Institutions and funders could address this by recognising open science contributions in evaluation, providing financial support for open access and data stewardship, and allowing flexibility in contracts or timelines to accommodate open science requirements.

Third, misconceptions should be addressed directly. Concerns about scooping or misuse were not prominent in this study, suggesting that these risks may be overstated relative to other barriers. However, given the potential for self‐selection bias, perceptions of these risks may differ in the wider ecology and evolutionary biology community. Communicating this evidence may help reduce reluctance to share data and code among researchers with similar perspectives to those represented in our sample, although perceptions may differ across the wider ecology and evolutionary biology community.

Finally, open science beyond academia is increasingly important (UNESCO 2021). As AI tools make scientific outputs more accessible and reusable (Pei and Huang 2025; Hosseini et al. 2025), understanding how research is produced, shared, and validated becomes crucial for policymakers, non‐profits and the public (Winker et al. 2023). Transparency and openness can strengthen this feedback loop, improving trust in science while accelerating the translation of research into societal benefit, particularly in policy‐relevant domains (Cole et al. 2024; Winker et al. 2023). This reinforces that sustaining trust in science in an increasingly open and politicised research environment requires greater coordination and collaboration across stakeholders in the global knowledge system, including researchers, publishers, institutions, funders, policymakers and society at large, spanning the full pathway from knowledge creation to societal use (UNESCO 2021; Zagrodzka et al. 2024; Pei and Huang 2025).

### 4.6 Future Directions

Our survey, based on a broad outreach to organisations and individuals working in ecology and evolutionary biology, provides insights into engagement, attitudes and commitment toward open science. However, it has several limitations that if addressed, may increase the representativeness of our sample and strengthen inference. Despite distributing the survey as broadly as possible, we were unable to determine how many survey invitations were successfully delivered, how many were filtered to spam or whether organisations forwarded the invitation internally. Similarly, dissemination through public platforms (e.g., X, Slack groups, forums and email lists) likely reached only certain demographics, and language barriers may have further limited participation from international respondents. Consequently, our survey may overrepresent individuals who are already familiar with open science, and those who hold positive attitudes toward it, introducing potential self‐selection bias, which may lead to overestimation of engagement of favourable perceptions of open science.

Although many respondents reported familiarity with open science, it is possible that their understanding does not align with formal definitions, such as those articulated by UNESCO (2021). Furthermore, because familiarity, attitudes and practices were measured at the same time, the direction of the relationships observed between these variables cannot be determined. As a result, survey responses may not fully capture broader perspectives or practices within the research community. Low response numbers further limit generalisability and increase the likelihood that our findings reflect the views of more interested or familiar participants.

Participation may also have been constrained by the lack of incentives, which are known to improve response rates. Finally, representation across professional sectors was uneven, with universities and research institutions predominating and other groups underrepresented. While these patterns provide useful descriptive insights, they must be interpreted with caution, as the survey may preferentially capture perspectives of more accessible or engaged segments of the community.

Despite these limitations, the survey offers a preliminary overview of current practices and perceptions regarding open science in ecology and evolutionary biology. It highlights areas where further investigation, perhaps using complementary qualitative or targeted methods, could provide a more nuanced understanding of engagement, barriers, and attitudes across the broader research landscape. Additionally, expanding beyond ecology and evolutionary biology would also allow assessment of the generalisability of these patterns across disciplines.

## Author Contributions

**Zuzanna B. Zagrodzka:** conceptualization (equal), data curation (lead), formal analysis (lead), funding acquisition (equal), investigation (equal), methodology (equal), project administration (lead), software (equal), validation (equal), visualization (lead), writing – original draft (lead), writing – review and editing (equal). **Thomas F. Johnson:** conceptualization (equal), formal analysis (equal), investigation (supporting), methodology (equal), software (supporting), supervision (supporting), validation (equal), visualization (supporting), writing – original draft (supporting), writing – review and editing (equal). **Andrew P. Beckerman:** conceptualization (equal), formal analysis (supporting), funding acquisition (equal), methodology (supporting), project administration (supporting), supervision (lead), writing – original draft (supporting), writing – review and editing (supporting).

## Funding

This work is funded by a studentship (Z.B.Z.) allocated to the University of Sheffield from JISC (grant no. 5370) and the UK Reproducibility Network. The funders had no role in study design, data collection and analysis, decision to publish, or preparation of the manuscript.

## Disclosure

For the purpose of open access, the authors have applied a Creative Commons Attribution (CC BY) licence to any author‐accepted manuscript versions arising.

## Conflicts of Interest

The authors declare no conflicts of interest.

## Supporting information


**Supplementary Information S1.** Model results and confidence intervals (CIs) for all five models.
**Table S1.1** Posterior summaries from a Bayesian Beta Regression Model of Familiarity with Open Science.
**Table S1.2** Posterior summaries from a Bayesian Bernoulli Regression Model for Positive Perceptions of Open Science Terms.
**Table S1.3** Posterior summaries from a Bayesian Bernoulli Regression Model for Negative Perceptions of Open Science Terms.
**Table S1.4** Posterior summaries from a Bayesian Bernoulli Regression Model for Engagement with Open Science Practices.
**Table S1.5** Posterior summaries from a Bayesian Beta Regression Model for Agreement with UNESCO Open Science Statements.
**Figure S1.1** Posterior credible intervals.
**Figure S1.2** Posterior credible intervals for Model 2 (positive perceptions).
**Figure S1.3** Posterior credible intervals for Model 3 (negative perceptions).
**Figure S1.4** Posterior credible intervals for Model 4 (Open Science Practices).
**Figure S1.5** Posterior credible intervals for Model 5 (UNESCO Agreement).

## Data Availability

The data underlying the findings of this study contain potentially identifiable participant information. As participants did not consent to public sharing of their data, the anonymised dataset has been deposited in the University of Sheffield ORDA institutional repository under restricted access. Researchers who meet the criteria for access to confidential data and agree to preserve participant confidentiality may request access via the repository or by contacting the corresponding author. To support reproducibility of the analysis pipeline, a synthetic dataset and the associated R project have been deposited on Zenodo. The blank survey instrument and accompanying metadata have also been deposited in the University of Sheffield ORDA institutional repository (Zagrodzka 2024). Code availability: The analysis code used in this study has been deposited on Zenodo (Zagrodzka 2026).

## References

[ece374595-bib-0001] Abele‐Brehm, A. E. , M. Gollwitzer , U. Steinberg , and F. D. Schönbrodt . 2019. “Attitudes Toward Open Science and Public Data Sharing.” Social Psychology 50: 252–260. 10.1027/1864-9335/a000384.

[ece374595-bib-0002] Abramo, G. , C. A. D'Angelo , and G. Murgia . 2013. “Gender Differences in Research Collaboration.” Journal of Informetrics 7: 811–822. 10.1016/j.joi.2013.07.002.

[ece374595-bib-0003] Alderson, D. , L. Clarke , D. Schillereff , and E. Shuttleworth . 2023. “Navigating the Academic Ladder as an Early Career Researcher in Earth and Environmental Sciences.” Earth Surface Processes and Landforms 48: 475–486. 10.1002/esp.5497.

[ece374595-bib-0004] Arza, V. , and M. Fressoli . 2017. “Systematizing Benefits of Open Science Practices.” Information Services and Use 37: 463–474. 10.3233/ISU-170861.

[ece374595-bib-0005] Aubert Bonn, N. , and W. Pinxten . 2021. “Rethinking Success, Integrity, and Culture in Research (Part 1)—A Multi‐Actor Qualitative Study on Success in Science.” Research Integrity and Peer Review 6: 1. 10.1186/s41073-020-00104-0. PMC7807516 33441187

[ece374595-bib-0006] Bear, J. B. , and A. W. Woolley . 2011. “The Role of Gender in Team Collaboration and Performance.” Interdisciplinary Science Reviews 36: 146–153. 10.1179/030801811X13013181961473.

[ece374595-bib-0007] Berezko, O. , L. M. Palma Medina , G. Malaguarnera , et al. 2021. “Perspectives on Open Science and Scholarly Publishing: A Survey Study Focusing on Early Career Researchers in Europe [Version 1; Peer Review: 2 Approved With Reservations].” 10.12688/f1000research.74831.1.

[ece374595-bib-0008] Bürkner, P.‐C. 2017. “brms: An R Package for Bayesian Multilevel Models Using Stan.” Journal of Statistical Software 80: 1–28. 10.18637/jss.v080.i01.

[ece374595-bib-0009] Bürkner, P.‐C. 2018. “Advanced Bayesian Multilevel Modeling With the R Package brms.” R Journal 10: 395. 10.32614/RJ-2018-017.

[ece374595-bib-0010] Bürkner, P.‐C. 2021. “Bayesian Item Response Modeling in R With brms and Stan.” Journal of Statistical Software 100: 1–54. 10.18637/jss.v100.i05.

[ece374595-bib-0011] Cech, E. A. 2022. “The Intersectional Privilege of White Able‐Bodied Heterosexual Men in STEM.” Science Advances 8: eabo1558. 10.1126/sciadv.abo1558. PMC9200289 35704581

[ece374595-bib-0012] Christie, A. P. , T. B. White , P. A. Martin , et al. 2021. “Reducing Publication Delay to Improve the Efficiency and Impact of Conservation Science.” PeerJ 9: e12245. 10.7717/peerj.12245. PMC8519180 34721971

[ece374595-bib-0013] Chtena, N. , J. P. Alperin , E. Morales , et al. 2025. “Towards an Inclusive Open Science: Examining EDI and Public Participation in Policy Documents Across Europe and the Americas.” Royal Society Open Science 12: 240857. 10.1098/rsos.240857. PMC12046529 40322273

[ece374595-bib-0014] COARA . 2025. “About CoARA—Coalition for Advancing Research Assessment.” https://www.coara.org/.

[ece374595-bib-0015] Cole, N. L. , E. Kormann , T. Klebel , S. Apartis , and T. Ross‐Hellauer . 2024. “The Societal Impact of Open Science: A Scoping Review.” Royal Society Open Science 11: 240286. 10.1098/rsos.240286. PMC11296153 39100167

[ece374595-bib-0016] Cruz‐Castro, L. , D. K. Ginther , and L. Sanz‐Menéndez . 2023. “Chapter 17: Gender and Underrepresented Minorities Differences in Research Funding.” In Handbook of Public Funding of Research, 279–300. Edward Elgar Publishing. 10.4337/9781800883086.00025.

[ece374595-bib-0017] Cruz‐Castro, L. , and L. Sanz‐Menendez . 2021. “What Should Be Rewarded? Gender and Evaluation Criteria for Tenure and Promotion.” Journal of Informetrics 15: 101196. 10.1016/j.joi.2021.101196.

[ece374595-bib-0018] Dick, P. 2024. “Introduction: Rethinking Gender Inequalities in Organizations.” In Rethinking Gender Inequalities in Organizations, 1–21. Edward Elgar Publishing. 10.4337/9781802207385.

[ece374595-bib-0019] European Commission . 2026. “Open Science—European Commission.” https://rea.ec.europa.eu/open‐science_en.

[ece374595-bib-0020] Farré, L. , Y. Fawaz , L. González , and J. Graves . 2022. “Gender Inequality in Paid and Unpaid Work During Covid‐19 Times.” Review of Income and Wealth 68: 323–347. 10.1111/roiw.12563.

[ece374595-bib-0021] Farrow, R. 2025. “Open Science and Epistemic Equity: Opportunities and Challenges in the Contemporary Research Ecosystem.” BMC Research Notes 18: 504. 10.1186/s13104-025-07608-2. PMC12699889 41382220

[ece374595-bib-0022] Fears, R. , K. A. B. Abdullah , C. Canales‐Holzeis , et al. 2021. “Evidence‐Informed Policy for Tackling Adverse Climate Change Effects on Health: Linking Regional and Global Assessments of Science to Catalyse Action.” PLoS Medicine 18: e1003719. 10.1371/journal.pmed.1003719. PMC8330928 34283834

[ece374595-bib-0023] Ferguson, J. , R. Littman , G. Christensen , et al. 2023. “Survey of Open Science Practices and Attitudes in the Social Sciences.” Nature Communications 14: 5401. 10.1038/s41467-023-41111-1. PMC10480148 37669942

[ece374595-bib-0024] Gownaris, N. J. , K. Vermeir , M.‐I. Bittner , et al. 2022. “Barriers to Full Participation in the Open Science Life Cycle Among Early Career Researchers.” Data Science Journal 21: 21. 10.5334/dsj-2022-002.

[ece374595-bib-0025] Hadorn, S. , F. Sager , C. Mavrot , A. Malandrino , and J. Ege . 2022. “Evidence‐Based Policymaking in Times of Acute Crisis: Comparing the Use of Scientific Knowledge in Germany, Switzerland, and Italy.” Politische Vierteljahresschrift 63: 359–382. 10.1007/s11615-022-00382-x. PMC8977835 35399336

[ece374595-bib-0026] Holman, L. , D. Stuart‐Fox , and C. E. Hauser . 2018. “The Gender Gap in Science: How Long Until Women Are Equally Represented?” PLoS Biology 16: e2004956. 10.1371/journal.pbio.2004956. PMC5908072 29672508

[ece374595-bib-0027] Hopf, H. , A. Krief , G. Mehta , and S. A. Matlin . 2019. “Fake Science and the Knowledge Crisis: Ignorance Can Be Fatal.” Royal Society Open Science 6: 190161. 10.1098/rsos.190161. PMC6549953 31218057

[ece374595-bib-0028] Hosseini, M. , S. P. J. M. Horbach , K. Holmes , and T. Ross‐Hellauer . 2025. “Open Science at the Generative AI Turn: An Exploratory Analysis of Challenges and Opportunities.” Quantitative Science Studies 6: 22–45. 10.1162/qss_a_00337. PMC11928019 40124128

[ece374595-bib-0029] Huang, C.‐K. , C. Neylon , R. Hosking , et al. 2020. “Evaluating the Impact of Open Access Policies on Research Institutions.” eLife 9: e57067. 10.7554/eLife.57067. PMC7536542 32924933

[ece374595-bib-0030] International Association of Scientific, Technical & Medical Publishers . 2026. “Uptake of Open Access.” In STM Advancing Trusted Research [Internet]. International Association of Scientific, Technical & Medical Publishers. https://stm‐assoc.org/oa‐dashboard/oa‐dashboard‐2/uptake‐of‐open‐access/.

[ece374595-bib-0031] Ivimey‐Cook, E. R. , A. Sánchez‐Tójar , I. Berberi , et al. 2025. “From Policy to Practice: Progress Towards Data‐ and Code‐Sharing in Ecology and Evolution.” Proceedings of the Royal Society B: Biological Sciences 292: 20251394. 10.1098/rspb.2025.1394. PMC12440620 40957576

[ece374595-bib-0032] Kambouris, S. , D. P. Wilkinson , E. T. Smith , and F. Fidler . 2024. “Computationally Reproducing Results From Meta‐Analyses in Ecology and Evolutionary Biology Using Shared Code and Data.” PLoS One 19: e0300333. 10.1371/journal.pone.0300333. PMC10936784 38478503

[ece374595-bib-0033] Krauss, A. , L. Danús , and M. Sales‐Pardo . 2023. “Early‐Career Factors Largely Determine the Future Impact of Prominent Researchers: Evidence Across Eight Scientific Fields.” Scientific Reports 13: 18794. 10.1038/s41598-023-46050-x. PMC10620415 37914796

[ece374595-bib-0034] Lerback, J. C. , B. Hanson , and P. Wooden . 2020. “Association Between Author Diversity and Acceptance Rates and Citations in Peer‐Reviewed Earth Science Manuscripts.” Earth and Space Science 7: e2019EA000946. 10.1029/2019EA000946.

[ece374595-bib-0035] Link, J. M. 2015. “Publish or Perish… but Where? What Is the Value of Impact Factors?” Nuclear Medicine and Biology 42: 426–427. 10.1016/j.nucmedbio.2015.01.004. PMC4387074 25656434

[ece374595-bib-0036] Mabile, L. , H. Shmagun , C. Erdmann , A. Cambon‐Thomsen , M. Thomsen , and F. Grattarola . 2025. “Recommendations on Open Science Rewards and Incentives: Guidance for Multiple Stakeholders in Research.” Data Science Journal 24: 1–19. 10.5334/dsj-2025-015.

[ece374595-bib-0037] Macaluso, B. , V. Larivière , T. Sugimoto , and C. R. Sugimoto . 2016. “Is Science Built on the Shoulders of Women? A Study of Gender Differences in Contributorship.” Academic Medicine 91: 1136–1142. 10.1097/ACM.0000000000001261. 27276004

[ece374595-bib-0038] Mitchneck, B. , J. L. Smith , and M. Latimer . 2016. “A Recipe for Change: Creating a More Inclusive Academy.” Science 352: 148–149. 10.1126/science.aad8493. 27124441

[ece374595-bib-0039] Moss‐Racusin, C. A. , J. F. Dovidio , V. L. Brescoll , M. J. Graham , and J. Handelsman . 2012. “Science Faculty's Subtle Gender Biases Favor Male Students.” Proceedings of the National Academy of Sciences of the United States of America 109: 16474–16479. 10.1073/pnas.1211286109. PMC3478626 22988126

[ece374595-bib-0040] National Research Council of the National Academies . 2012. Using Science as Evidence in Public Policy, 13460. National Academies Press. 10.17226/13460.

[ece374595-bib-0041] Nguyen, V. M. , N. R. Haddaway , L. F. G. Gutowsky , et al. 2015. “How Long Is Too Long in Contemporary Peer Review? Perspectives From Authors Publishing in Conservation Biology Journals.” PLoS One 10: e0132557. 10.1371/journal.pone.0132557. PMC4533968 26267491

[ece374595-bib-0042] Ni, C. , E. Smith , H. Yuan , V. Larivière , and C. R. Sugimoto . 2021. “The Gendered Nature of Authorship.” Science Advances 7: eabe4639. 10.1126/sciadv.abe4639. PMC8442765 34516891

[ece374595-bib-0043] Nosek, B. A. , G. Alter , G. C. Banks , et al. 2015. “Promoting an Open Research Culture.” Science 348: 1422–1425. 10.1126/science.aab2374. PMC4550299 26113702

[ece374595-bib-0044] O'Donnell, R. P. , S. R. Supp , and S. M. Cobbold . 2010. “Hindrance of Conservation Biology by Delays in the Submission of Manuscripts.” Conservation Biology 24: 615–620. 10.1111/j.1523-1739.2009.01424.x. 20067489

[ece374595-bib-0045] Olesk, A. , E. Kaal , and K. Toom . 2019. “The Possibilities of Open Science for Knowledge Transfer in the Science‐Policy Interface.” Journal of Science Communication 18: A03. 10.22323/2.18030203.

[ece374595-bib-0046] Oliver, K. , S. Innvar , T. Lorenc , J. Woodman , and J. Thomas . 2014. “A Systematic Review of Barriers to and Facilitators of the Use of Evidence by Policymakers.” BMC Health Services Research 14: 2. 10.1186/1472-6963-14-2. PMC3909454 24383766

[ece374595-bib-0047] Ollé, C. , A. López‐Borrull , R. Melero , J.‐J. Boté‐Vericad , J.‐M. Rodríguez‐Gairín , and E. Abadal . 2023. “Habits and Perceptions Regarding Open Science by Researchers From Spanish Institutions.” PLoS One 18: e0288313. 10.1371/journal.pone.0288313. PMC10343031 37440550

[ece374595-bib-0048] Page, M. J. , P.‐Y. Nguyen , D. G. Hamilton , et al. 2022. “Data and Code Availability Statements in Systematic Reviews of Interventions Were Often Missing or Inaccurate: A Content Analysis.” Journal of Clinical Epidemiology 147: 1–10. 10.1016/j.jclinepi.2022.03.003. 35278609

[ece374595-bib-0049] Pardo Martínez, C. I. , and A. C. Poveda . 2018. “Knowledge and Perceptions of Open Science Among Researchers—A Case Study for Colombia.” Information 9: 292. 10.3390/info9110292.

[ece374595-bib-0050] Pei, G. , and H. Huang . 2025. “Open Science Falling Behind in the Era of Artificial Intelligence.” Frontiers in Research Metrics and Analytics 10: 10. 10.3389/frma.2025.1595824. PMC12328315 40778108

[ece374595-bib-0051] Pethick, S. , M. N. Wass , and M. Michaelis . 2025. “Is There a Reproducibility Crisis? On the Need for Evidence‐Based Approaches.” International Studies in the Philosophy of Science 38: 287–303. 10.1080/02698595.2025.2538937.

[ece374595-bib-0052] Phelps, R. 2022. “Challenging the Academic Publisher Oligopoly.” *The James G. Martin Center for Academic Renewal* [Internet]. James G. Martin Center for Academic Renewal, November 16, 2022. https://jamesgmartin.center/2022/11/challenging‐the‐academic‐publisher‐oligopoly/.

[ece374595-bib-0053] Piasna, A. , and J. Drahokoupil . 2017. “Gender Inequalities in the New World of Work.” Transfer: European Review of Labour and Research 23: 313–332. 10.1177/1024258917713839.

[ece374595-bib-0054] Rawat, S. , and S. Meena . 2014. “Publish or Perish: Where Are We Heading?” Journal of Research in Medical Sciences 19: 87–89. PMC3999612 24778659

[ece374595-bib-0055] Régner, I. , C. Thinus‐Blanc , A. Netter , T. Schmader , and P. Huguet . 2019. “Committees With Implicit Biases Promote Fewer Women When They Do Not Believe Gender Bias Exists.” Nature Human Behaviour 3: 1171–1179. 10.1038/s41562-019-0686-3. 31451735

[ece374595-bib-0056] Reichmann, S. , and B. Wieser . 2022. “Open Science at the Science–Policy Interface: Bringing in the Evidence?” Health Research Policy and Systems 20: 70. 10.1186/s12961-022-00867-6. PMC9208144 35725491

[ece374595-bib-0057] Riedl, C. , Y. J. Kim , P. Gupta , T. W. Malone , and A. W. Woolley . 2021. “Quantifying Collective Intelligence in Human Groups.” Proceedings of the National Academy of Sciences of the United States of America 118: e2005737118. 10.1073/pnas.2005737118. PMC8166150 34001598

[ece374595-bib-0058] Roche, D. G. , R. E. O'Dea , K. A. Kerr , et al. 2022. “Closing the Knowledge‐Action Gap in Conservation With Open Science.” Conservation Biology 36: e13835. 10.1111/cobi.13835. PMC9300006 34476839

[ece374595-bib-0059] Ross, M. B. , B. M. Glennon , R. Murciano‐Goroff , E. G. Berkes , B. A. Weinberg , and J. I. Lane . 2022. “Women Are Credited Less in Science Than Men.” Nature 608: 135–145. 10.1038/s41586-022-04966-w. PMC9352587 35732238

[ece374595-bib-0060] Rowley, J. , L. Sbaffi , M. Sugden , and A. Gilbert . 2022. “Factors Influencing Researchers' Journal Selection Decisions.” Journal of Information Science 48: 321–335. 10.1177/0165551520958591.

[ece374595-bib-0061] Salerno, P. E. , M. Páez‐Vacas , J. M. Guayasamin , and J. L. Stynoski . 2019. “Male Principal Investigators (Almost) Don't Publish With Women in Ecology and Zoology.” PLoS One 14: e0218598. 10.1371/journal.pone.0218598. PMC6583967 31216351

[ece374595-bib-0062] Soeharjono, S. , and D. G. Roche . 2021. “Reported Individual Costs and Benefits of Sharing Open Data Among Canadian Academic Faculty in Ecology and Evolution.” Bioscience 71: 750–756. 10.1093/biosci/biab024.

[ece374595-bib-0063] SPARC . 2026. “Browse Publication and Data Sharing Requirements by Federal Agency.” https://researchsharing.sparcopen.org/.

[ece374595-bib-0064] Suazo‐Galdames, I. C. , M. Saracostti , and A. M. Chaple‐Gil . 2025. “Scientific Evidence and Public Policy: A Systematic Review of Barriers and Enablers for Evidence‐Informed Decision‐Making.” Frontiers in Communication 10: 1632305. 10.3389/fcomm.2025.1632305.

[ece374595-bib-0065] Tedersoo, L. , R. Küngas , E. Oras , et al. 2021. “Data Sharing Practices and Data Availability Upon Request Differ Across Scientific Disciplines.” Scientific Data 8: 192. 10.1038/s41597-021-00981-0. PMC8381906 34315906

[ece374595-bib-0066] The Lancet Regional Health – Western Pacific . 2022. “The Intersection of Science and Governance.” Lancet Regional Health – Western Pacific 27: 100625. 10.1016/j.lanwpc.2022.100625. PMC9614182 36320421

[ece374595-bib-0067] Thibault, R. T. , O. B. Amaral , F. Argolo , A. E. Bandrowski , R. D. Alexandra , and N. I. Drude . 2023. “Open Science 2.0: Towards a Truly Collaborative Research Ecosystem.” PLoS Biology 21: e3002362. 10.1371/journal.pbio.3002362. PMC10617723 37856538

[ece374595-bib-0068] UNESCO . 2021. UNESCO Recommendation on Open Science. UNESCO. https://unesdoc.unesco.org/ark:/48223/pf0000379949.

[ece374595-bib-0069] UNESCO . 2022. Developing Policies for Open Science—UNESCO Digital Library. UNESCO. 10.54677/VHNY8608.

[ece374595-bib-0070] van der Lee, R. , and N. Ellemers . 2015. “Gender Contributes to Personal Research Funding Success in The Netherlands.” Proceedings of the National Academy of Sciences of the United States of America 112: 12349–12353. 10.1073/pnas.1510159112. PMC4603485 26392544

[ece374595-bib-0071] Vial, A. C. , and C. M. Cowgill . 2022. “Heavier Lies Her Crown: Gendered Patterns of Leader Emotional Labor and Their Downstream Effects.” Frontiers in Psychology 13: 849566. 10.3389/fpsyg.2022.849566. PMC9465331 36106035

[ece374595-bib-0072] Winker, M. A. , T. Bloom , S. Onie , and J. Tumwine . 2023. “Equity, Transparency, and Accountability: Open Science for the 21st Century.” Lancet 402: 1206–1209. 10.1016/S0140-6736(23)01575-1. 37805196

[ece374595-bib-0073] Witteman, H. O. , M. Hendricks , S. Straus , and C. Tannenbaum . 2019. “Are Gender Gaps due to Evaluations of the Applicant or the Science? A Natural Experiment at a National Funding Agency.” Lancet 393: 531–540. 10.1016/S0140-6736(18)32611-4. 30739688

[ece374595-bib-0074] Wróblewska, M. N. , C. Balaban , G. Derrick , and P. Benneworth . 2024. “The Conflict of Impact for Early Career Researchers Planning for a Future in the Academy.” Research Evaluation 33: rvad024. 10.1093/reseval/rvad024.

[ece374595-bib-0075] Zagrodzka, Z. B. 2024. Survey Metadata and Blank Survey of the Research Project: “Unlocking the Potential of Open Science for Knowledge Mobilisation”. University of Sheffield; 2026. 10.15131/shef.data.31558075.v1.

[ece374595-bib-0076] Zagrodzka, Z. B. 2026. “Characteristics‐of‐Professionals‐Involved‐in‐Open‐Science‐in‐Ecology‐and‐Evolution.” Zenodo. 10.5281/zenodo.18889105. PMC13727283 42974635

[ece374595-bib-0077] Zagrodzka, Z. B. , T. F. Johnson , and A. P. Beckerman . 2024. “Accelerating the Open Research Agenda to Solve Global Challenges.” Ecology and Evolution 14: e10887. 10.1002/ece3.10887. PMC10830346 38304275

