# Supplementary material for: Characteristics of Professionals Involved in Open Science in Ecology and Evolution
Source: Ecol Evol. 2026 Nov 2;16(11):e74595. doi: 10.1002/ece3.74595 (PMC13727283; doi:10.1002/ece3.74595)
Supplement: Supplementary file 1 [file ECE3-16-e74595-s001.pdf]

Supporting Information S1. Model Results and CIs

for

Characteristics of Professionals Involved in Open Science in Ecology and Evolution

Zuzanna B. Zagrodzka*, Thomas F. Johnson, Andrew P. Beckerman

*z.zagrodzka@sheffield.ac.uk

Table of Contents:

About the Supporting Information S1 2
Model 1 2
Model 1: Bayesian Model Results 2
Table S1.1. Posterior Summaries from a Bayesian Beta Regression Model of Familiarity with Open Science 3
Model 1: Posterior Credible Intervals 3
Figure S1.1. Posterior Credible Intervals 4
Model 2 5
Model 2: Bayesian Model Results 5
Table S1.2. Posterior Summaries from a Bayesian Bernoulli Regression Model for Positive Perceptions of Open Science Terms 6
Model 2: Posterior Credible Intervals 6
Figure S1.2. Posterior Credible Intervals for Model 2 (Positive Perceptions) 7
Model 3 8
Model 3: Bayesian Model Results (Negative Perceptions) 8
Table S1.3. Posterior Summaries from a Bayesian Bernoulli Regression Model for Negative Perceptions of Open Science Terms 9
Model 3: Posterior Credible Intervals 9
Figure S1.3. Posterior Credible Intervals for Model 3 (Negative Perceptions) 10
Model 4 11
Model 4: Bayesian Model Results 11
Table S1.4. Posterior Summaries from a Bayesian Bernoulli Regression Model for Engagement with Open Science Practices 12
Model 4: Posterior Credible Intervals 12
Figure S1.4. Posterior Credible Intervals for Model 4 (Open Science Practices) 13
Model 5 14
Model 5: Bayesian Model Results 14
Table S1.5. Posterior Summaries from a Bayesian Beta Regression Model for Agreement with UNESCO Open Science Statements 15
Model 5: Posterior Credible Intervals 15
Figure S1.5. Posterior Credible Intervals for Model 5 (UNESCO Agreement) 16

About the Supporting Information S1

This supporting information (S1) contains Bayesian Model Results and Posterior Credible Intervals for all models we fitted to the survey data and presented in a paper titled: "Who's Behind the Push for Openness? Understanding the Actors in the Open Science Landscape in Ecology and Evolution."

Model 1

Sections "Model 1" provide supporting information for the Bayesian model fitted to answer the following research question:

i) Does the effect of the years of experience on familiarity on open science vary by stakeholder group or gender?

Model 1: Bayesian Model Results

The table S1.1 contains the fixed-effect estimates from a Bayesian Beta regression model predicting respondents' **familiarity** with open science. The model includes **stakeholder group**, **gender**, and **years of professional experience** as predictors, along with interaction terms between stakeholder group and experience, and gender and experience.

- **Stakeholder group:** "Knowledge Creator" serves as the reference category. Other groups are "Knowledge Mobiliser in Academia" and "Knowledge Mobiliser outside Academia".
- **Gender**: "Female" serves as the reference category. The model includes the main effect of being male and its interaction with years of experience.
- **Years of experience:** Continuous variable representing the number of years of professional experience, modeled both as a main effect and in interactions with stakeholder group and gender to capture differential slopes.
- **Coding:** Treatment coding was used for categorical predictors, such that all coefficients are interpreted relative to the reference levels.
- **Priors:** Weakly informative priors were applied: normal (0, 2.5) for all slope and intercept parameters, and exponential for the Beta precision parameter.
- **Estimates**: For each fixed effect, the table provides the posterior mean estimate, standard error, and 95% credible interval. Positive estimates indicate higher predicted familiarity with open science relative to the reference category, while negative estimates indicate lower predicted familiarity. Interaction terms indicate how the effect of years of experience varies by stakeholder group or gender.
- **Table formatting:** The table highlights terms in bold, presents credible intervals for precision, and is structured for clear interpretation of both main and interaction effects.

The model accounts for missing data using multiple imputation, and results reflect the posterior distribution of parameters after combining information across imputed datasets.

Model 1 - Question 1

Term	Estimate	Est.Error	l-95% CI	u-95% CI
**Intercept**	0.806	0.101	0.605	1.003
**Knowledge Mobiliser in Academia**	0.432	0.195	0.053	0.819
**Knowledge Mobiliser outside Academia**	-0.362	0.228	-0.806	0.089
**Years of Experience**	0.030	0.015	0.002	0.059
**Gender (Male)**	0.112	0.135	-0.151	0.378
**Knowledge Mobiliser in Academia:Years of Experience**	-0.020	0.027	-0.072	0.033
**Knowledge Mobiliser outside Academia:Years of Experience**	-0.020	0.030	-0.080	0.040
**Years of Experience:Gender (Male)**	-0.017	0.019	-0.055	0.021

Table S1.1. Posterior Summaries from a Bayesian Beta Regression Model of Familiarity with Open Science.

Model 1: Posterior Credible Intervals

Posterior samples were extracted for each coefficient, and 95%, 80%, and 50% credible intervals were computed to visualise uncertainty in model. The intervals correspond to the following quantiles of the posterior distribution:

- 95% CI: 2.5% – 97.5%
- 80% CI: 10% – 90%
- 50% CI: 25% – 75%

For each parameter, the median (50th percentile) and these intervals were calculated, allowing layered visualisation of parameter uncertainty.

Posterior estimates were grouped into three categories for plotting (see Figure S1.1):
A) Intercept. The model baseline, representing the reference levels.
B) Continuous variable. Years of experience. The continuous predictor, including main effects and interactions.
C) Categorical variables. Stakeholder group and gender effects, including interactions with experience.

For each group, posterior medians are shown as points, with error bars representing the 50%, 80%, and 95% credible intervals. A vertical dashed line at $\beta = 0$ highlights the null (no-effect) value for regression coefficients on the linear prediction scale. This highlights both main effects and interactions.

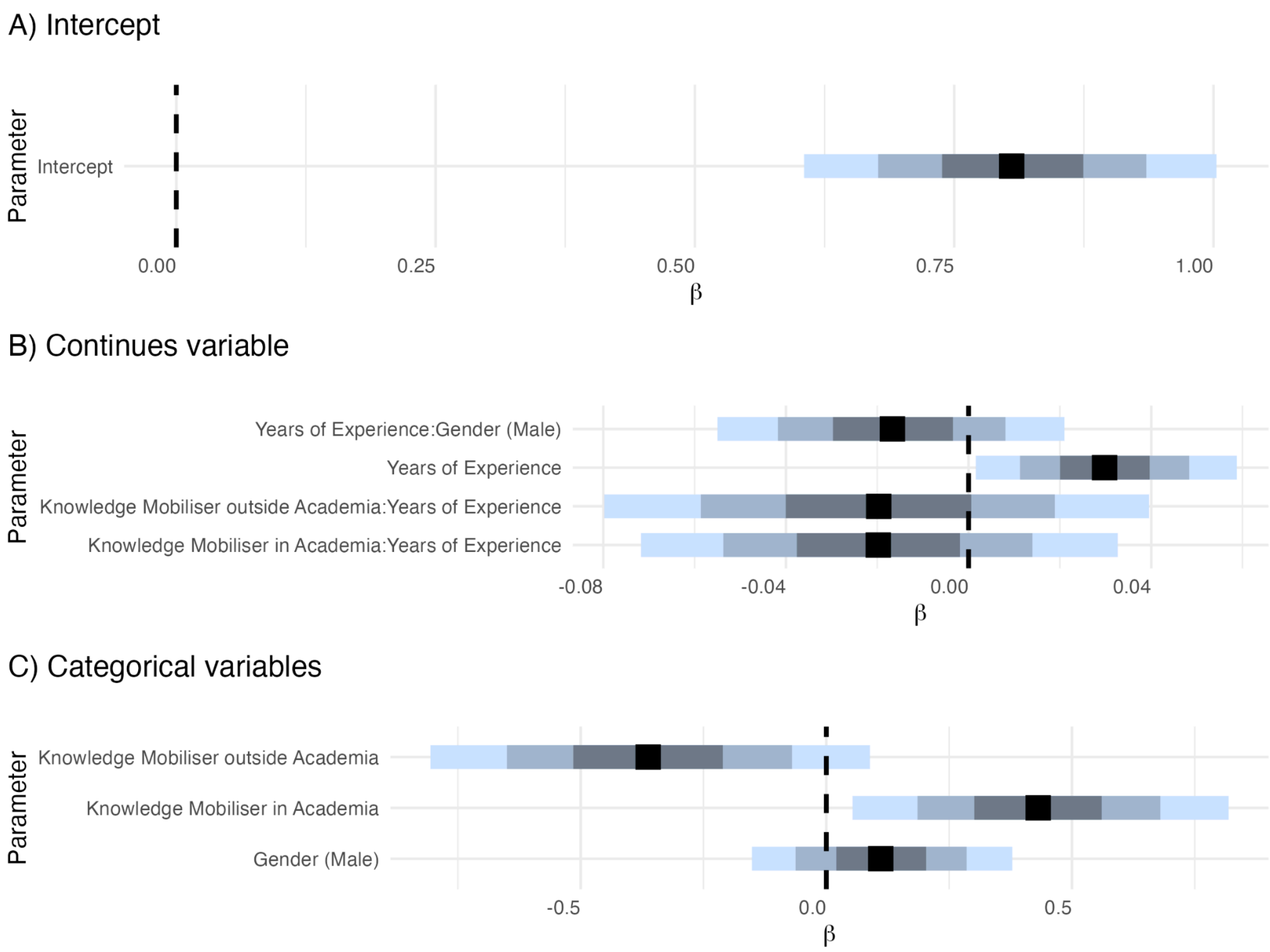

Figure S1.1. Posterior Credible Intervals.

Model 2

Sections “Model 2” provides supporting information for the Bayesian model fitted to answer the following research question:

ii) Do years of experience and familiarity influence positive and negative associations with open science, and do these effects vary by stakeholder group or gender?

Model 2: Bayesian Model Results

The table S1.2 presents the fixed-effect estimates from a Bayesian Bernoulli regression model predicting positive perceptions of open science terms. The model includes stakeholder group, gender, years of professional experience, and familiarity with open science as predictors, along with interaction terms between stakeholder group and years of experience, and gender and years of experience.

Random intercepts were included for respondents and for open science terms to account for repeated observations and term-specific variability.

- **Outcome:** Binary indicator of a positive perception of an open science term.
- **Stakeholder group:** “Knowledge Creator” serves as the reference category. Other groups include “Knowledge Mobiliser in Academia” and “Knowledge Mobiliser outside Academia”.
- **Gender:** “Female” serves as the reference category. The model includes the main effect of being male and its interaction with years of experience.
- **Years of experience:** A continuous variable representing professional experience. Values were centred at 10 years to facilitate interpretation of the intercept as the expected log-odds of a positive perception for a moderately experienced professional in the reference categories.
- **Familiarity with open science:** Included as a fixed-effect predictor to assess whether greater familiarity is associated with an increased likelihood of expressing positive perceptions.
- **Coding:** Categorical predictors were coded using treatment coding, such that all coefficients are interpreted relative to the reference levels.
- **Priors:** Weakly informative priors were applied: normal (0, 2.5) for fixed effects, and Student-t (3, 0, 1) priors for the standard deviations of the random intercepts.
- **Estimates:** For each fixed effect, the table reports the posterior mean estimate, standard error, and 95% credible interval. Coefficients are reported on the log-odds (logit) scale. Positive estimates indicate higher odds of expressing a positive perception, whereas negative estimates indicate lower odds. Interaction terms indicate whether the effect of years of experience varies by stakeholder group or gender.
- **Missing data:** The model accounts for missing data using multiple imputation, and results reflect the posterior distribution after combining information across imputations.

Model 2 - Question 2 (Positive)				
**Term**	**Estimate**	**Est.Error**	**l-95% CI**	**u-95% CI**
**Knowledge Creator**	-0.080	0.616	-1.302	1.111
**Knowledge Mobiliser in Academia**	-0.046	0.669	-1.383	1.248
**Knowledge Mobiliser outside Academia**	0.317	0.653	-0.977	1.585
**Years of Experience**	-0.054	0.021	-0.094	-0.013
**Gender (Male)**	-0.217	0.193	-0.596	0.165
**Familiarity OS**	1.179	0.403	0.392	1.964
**Knowledge Mobiliaser in Academia:Years of Experience**	0.064	0.039	-0.013	0.141
**Knowledge Mobiliaser outside Academia:Years of Experience**	0.090	0.042	0.007	0.173
**Years of Experience:Gender (Male)**	0.017	0.028	-0.039	0.071

Table S1.2. Posterior Summaries from a Bayesian Bernoulli Regression Model for Positive Perceptions of Open Science Terms.

Model 2: Posterior Credible Intervals

Posterior samples were extracted for each model coefficient, and 95%, 80%, and 50% credible intervals were computed to visualise uncertainty in the parameter estimates. The intervals correspond to the following posterior quantiles:

- 95% credible interval: 2.5% – 97.5%
- 80% credible interval: 10% – 90%
- 50% credible interval: 25% – 75%

For each parameter, the posterior median (50th percentile) and these intervals were calculated, allowing layered visualisation of uncertainty across different credibility levels.

Posterior estimates were grouped into three categories for plotting (see Figure S1.3):
A) Intercepts. including random-effect standard deviations for respondents and terms;
B) Continuous variable. Years of experience, including main effects and interactions;
C) Categorical variables. Including stakeholder group, gender, familiarity with open science, and interaction terms.

For each parameter, posterior medians are shown as points, with horizontal bars representing the 50%, 80%, and 95% credible intervals. A vertical dashed line at $\beta = 0$ indicates the null (no-effect) value on the log-odds scale, facilitating visual assessment of the direction and strength of effects across parameters.

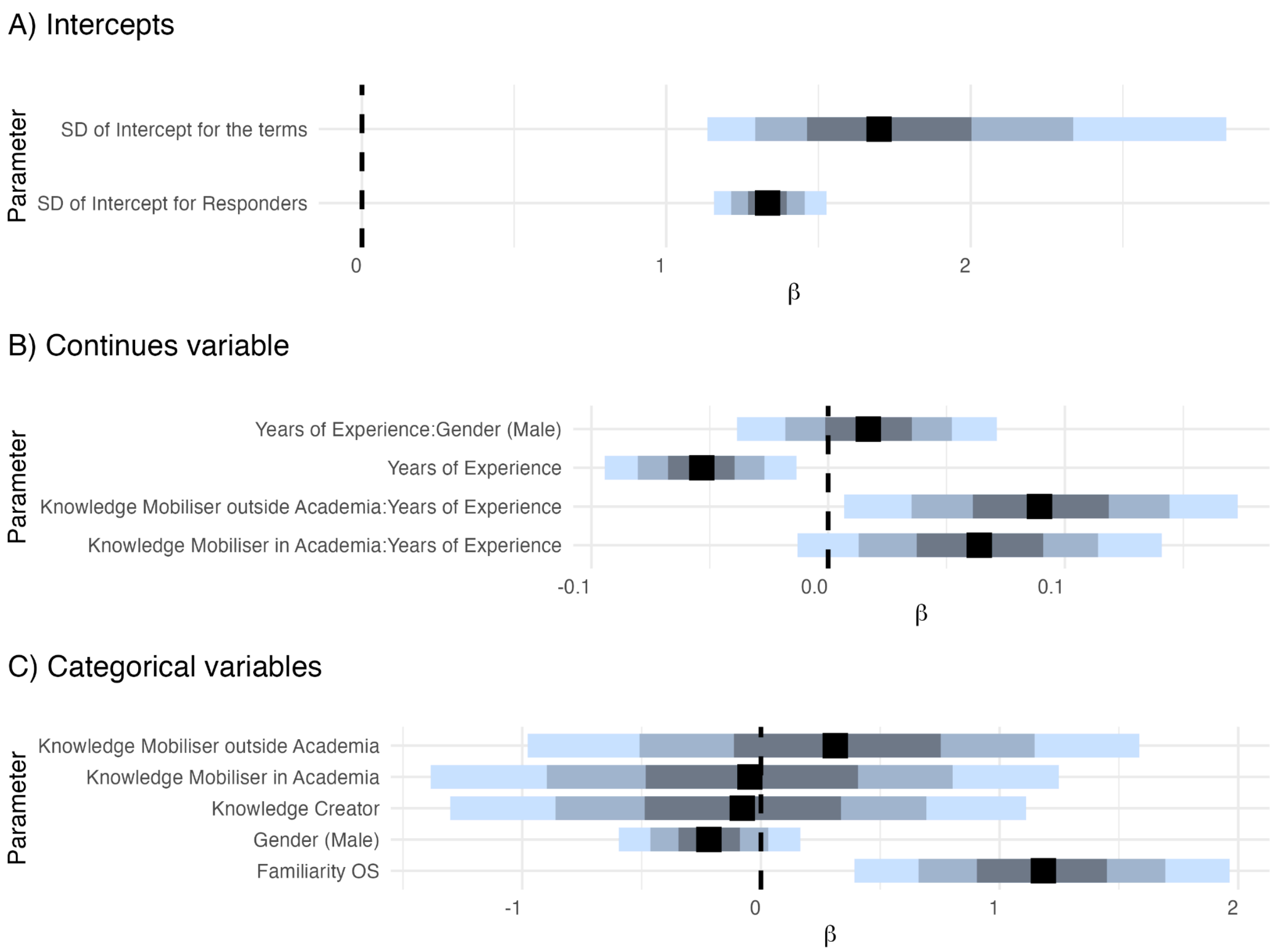

Figure S1.2. Posterior Credible Intervals for Model 2 (Positive Perceptions)

Model 3

Sections "Model 3" provide supporting information for the Bayesian model fitted to answer the following research question:

ii) Do years of experience and familiarity influence positive and negative associations with open science, and do these effects vary by stakeholder group or gender?

Model 3: Bayesian Model Results (Negative Perceptions)

The table S1.3 presents the fixed-effect estimates from a Bayesian Bernoulli regression model predicting negative perceptions of open science terms. The model includes stakeholder group, gender, years of professional experience, and familiarity with open science as predictors, along with interaction terms between stakeholder group and years of experience, and gender and years of experience.

Random intercepts were included for respondents and for open science terms to account for repeated observations and term-specific variability.

- **Outcome:** Binary indicator of a negative perception of an open science term.
- **Stakeholder group**: "Knowledge Creator" serves as the reference category. Other groups include "Knowledge Mobiliser in Academia" and "Knowledge Mobiliser outside Academia".
- **Gender:** "Female" serves as the reference category. The model includes the main effect of being male and its interaction with years of experience.
- **Years of experience:** A continuous variable representing professional experience, included as a main effect and in interactions with stakeholder groups and gender to assess whether experience-related effects differ across groups.
- **Familiarity with open science:** Included as a fixed-effect predictor to assess whether familiarity is associated with the likelihood of expressing negative perceptions.
- **Coding:** Categorical predictors were coded using treatment coding, such that all coefficients are interpreted relative to the reference levels.
- Priors: Weakly informative priors were applied: normal (0, 2.5) for fixed effects, and Student-t (3, 0, 1) priors for the standard deviations of the random intercepts.
- **Estimates:** For each fixed effect, the table reports the posterior mean estimate, standard error, and 95% credible interval. Coefficients are reported on the log-odds (logit) scale. Positive estimates indicate higher odds of expressing a negative perception, whereas negative estimates indicate lower odds. Interaction terms indicate whether the effect of years of experience varies by stakeholder group or gender.
- **Missing data:** The model accounts for missing data using multiple imputation, and results reflect the posterior distribution after combining information across imputations.

Model 3 - Question 2 (Negative)

Term	Estimate	Est.Error	l-95% CI	u-95% CI
**Knowledge Creator**	-0.975	0.615	-2.176	0.286
**Knowledge Mobiliser in Academia**	-1.217	0.661	-2.501	0.132
**Knowledge Mobiliser outside Academia**	-1.108	0.650	-2.397	0.208
**Years of Experience**	0.056	0.020	0.018	0.095
**Gender (Male)**	-0.402	0.182	-0.762	-0.047
**Familiarity OS**	-0.229	0.381	-0.984	0.519
**Knowledge Mobiliser in Academia:Years of Experience**	-0.114	0.038	-0.189	-0.038
**Knowledge Mobiliser outside Academia:Years of Experience**	-0.005	0.039	-0.083	0.073
**Years of Experience:Gender (Male)**	-0.027	0.026	-0.078	0.024

Table S1.3. Posterior Summaries from a Bayesian Bernoulli Regression Model for Negative Perceptions of Open Science Terms

Model 3: Posterior Credible Intervals

Posterior samples were extracted for each model coefficient, and 95%, 80%, and 50% credible intervals (CI) were computed to visualise uncertainty in the parameter estimates. The intervals correspond to the following posterior quantiles:

- 95% CI: 2.5% - 97.5%
- 80% CI: 10% - 90%
- 50% CI: 25% - 75%

For each parameter, the posterior median (50th percentile) and these intervals were calculated, allowing layered visualisation of uncertainty across different credibility levels.

Posterior estimates were grouped into three categories for plotting (see Figure S1.3):
A) **Intercepts.** Representing baseline log-odds for the reference categories and random-effect structure;
B) **Continuous variable**. Years of experience. Including main effects and interactions;
C) **Categorical variables.** Including stakeholder group, gender, familiarity with open science, and interaction terms.

For each parameter, posterior medians are shown as points, with horizontal bars representing the 50%, 80%, and 95% credible intervals. A vertical dashed line at $\beta = 0$ indicates the null (no-effect) value on the log-odds scale, facilitating visual assessment of the direction and strength of effects across parameters.

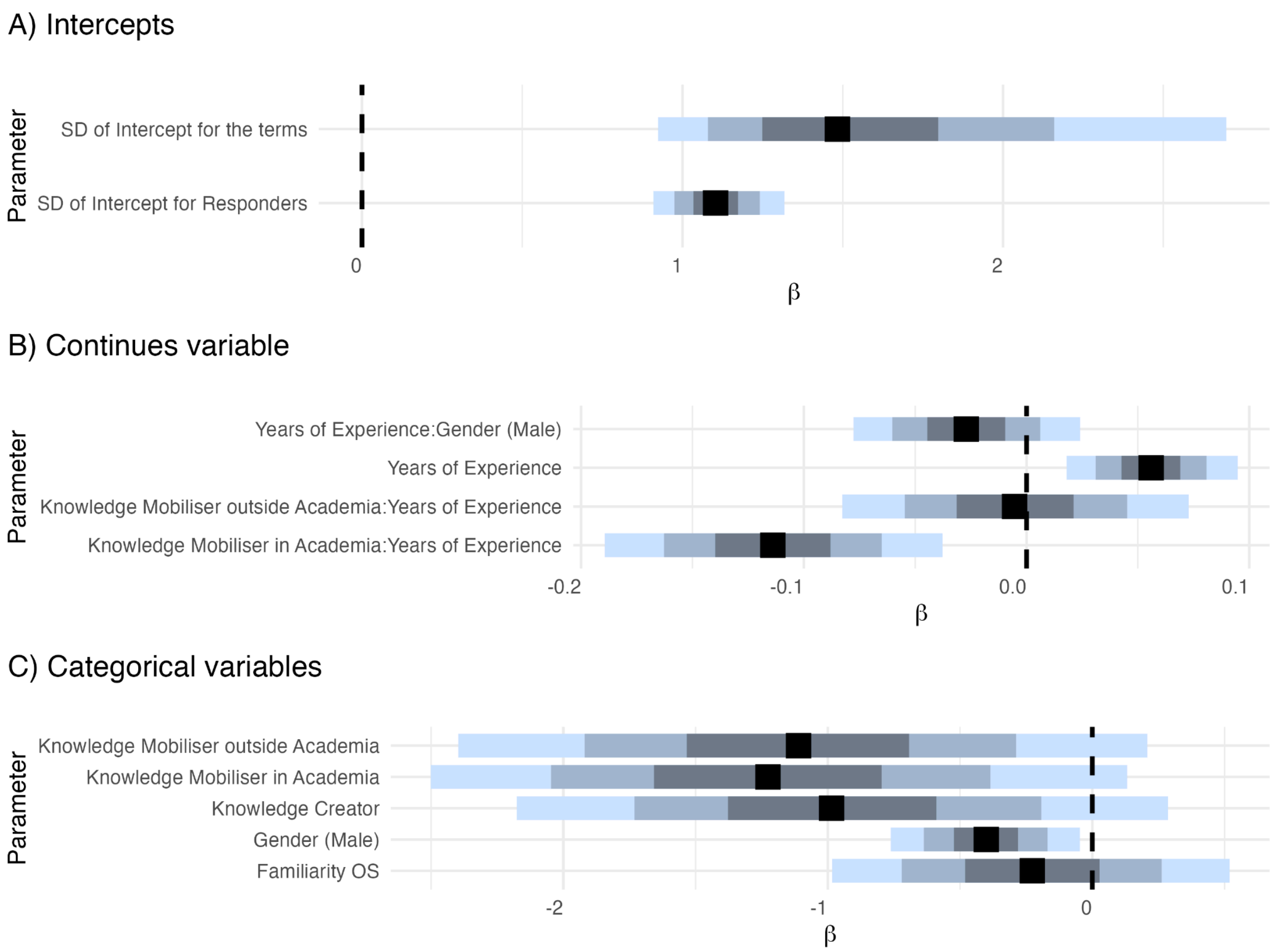

Figure S1.3. Posterior Credible Intervals for Model 3 (Negative Perceptions)

Model 4

Sections "Model 4" provide supporting information for the Bayesian model fitted to answer the following research question:

iii) Does the effect of years of experience and familiarity on open science practices vary by gender?

Model 4: Bayesian Model Results

Table S1.4 presents the fixed-effect estimates from a Bayesian Bernoulli regression model predicting engagement with open science practices among respondents affiliated with research institutes. The model includes gender, years of professional experience, and familiarity with open science as predictors, along with an interaction between gender and years of experience.

Random intercepts were included for respondents and for open science practices to account for repeated observations and practice-specific variability.

- **Outcome:** Binary indicator of engagement with an open science practice.
- **Gender:** "Female" serves as the reference category. The model includes the main effect of being male and its interaction with years of experience.
- **Years of experience:** A continuous variable representing professional experience, included as a main effect and in interaction with gender to assess whether experience-related effects differ by gender.
- **Familiarity with open science:** Included as a fixed-effect predictor to assess whether familiarity is associated with engagement in open science practices.
- **Coding:** Categorical predictors were coded using treatment coding, such that all coefficients are interpreted relative to the reference level.
- **Priors:** Weakly informative priors were applied: normal (0, 2.5) for fixed effects, and Student-t (3, 0, 1) priors for the standard deviations of the random intercepts.
- **Estimates:** For each fixed effect, the table reports the posterior mean estimate, standard error, and 95% credible interval. Coefficients are reported on the log-odds (logit) scale. Positive estimates indicate higher odds of engaging in open science practices, whereas negative estimates indicate lower odds. Interaction terms indicate whether the effect of years of experience varies by gender.
- **Missing data:** The model accounts for missing data using multiple imputation, and results reflect the posterior distribution after combining information across imputations.

Model 4 - Question 3

Term	Estimate	Est.Error	l-95% CI	u-95% CI
**Familiarity OS**	0.158	0.056	0.050	0.268
**Gender (Female)**	-1.805	0.633	-3.044	-0.542
**Gender (Male)**	-1.644	0.646	-2.893	-0.351
**Years of Experience**	0.014	0.025	-0.036	0.062
**Gender (Male):Years of Experience**	-0.054	0.036	-0.123	0.017

Table S1.4. Posterior Summaries from a Bayesian Bernoulli Regression Model for Engagement with Open Science Practices

Model 4: Posterior Credible Intervals

Posterior samples were extracted for each model coefficient, and 95%, 80%, and 50% credible intervals (CI) were computed to visualise uncertainty in the parameter estimates. The intervals correspond to the following posterior quantiles:

- 95% CI: 2.5% – 97.5%
- 80% CI: 10% – 90%
- 50% CI: 25% – 75%

For each parameter, the posterior median (50th percentile) and these intervals were calculated, allowing layered visualisation of uncertainty across different credibility levels.

Posterior estimates were grouped into three categories for plotting (see Figure S1.4):
A) **Intercepts.** Representing baseline log-odds for the reference category and random-effect structure;
B) **Continuous variable.** Years of experience, including the main effect and interaction with gender;
C) **Categorical variables.** Including gender and familiarity with open science.

For each parameter, posterior medians are shown as points, with horizontal bars representing the 50%, 80%, and 95% credible intervals. A vertical dashed line at $\beta = 0$ indicates the null (no-effect) value on the log-odds scale, facilitating visual assessment of the direction and strength of effects across parameters.

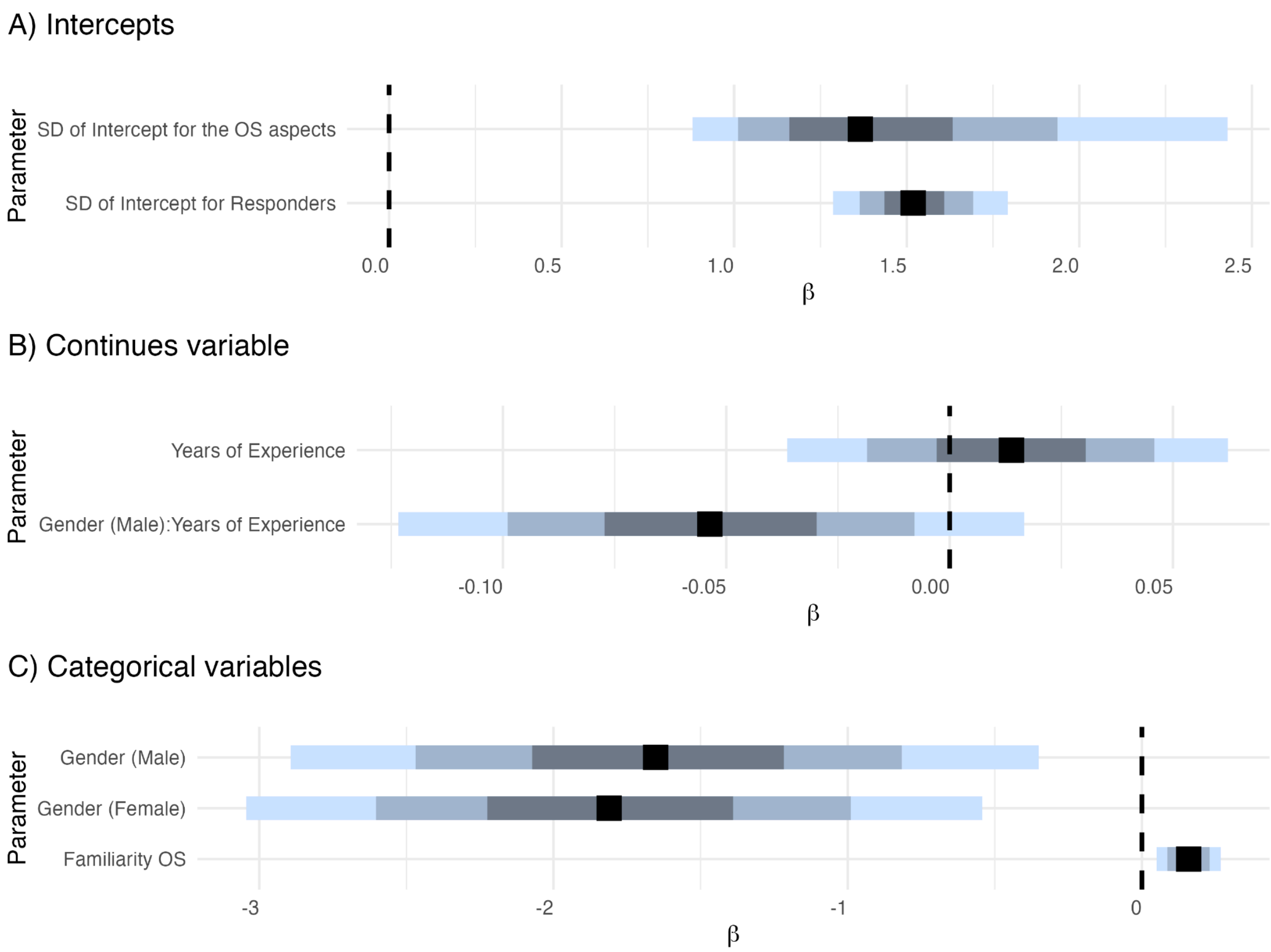

Figure S1.4. Posterior Credible Intervals for Model 4 (Open Science Practices)

Model 5

Sections "Model 5" provide supporting information for the Bayesian model fitted to answer the following research question:

iv) Does the effect of years of experience and familiarity on agreement with UNESCO open science statements vary by gender or stakeholder group?

Model 5: Bayesian Model Results

Table S1.5 presents the fixed-effect estimates from a Bayesian Beta regression model predicting agreement with rephrased statements derived from the UNESCO Recommendations on Open Science (2021). These statements were adapted for survey use to reflect core principles and recommendations of the UNESCO framework rather than verbatim policy text. The model includes stakeholder group, gender, years of professional experience, and familiarity with open science as predictors, along with interaction terms between stakeholder group and years of experience, and gender and years of experience.

Random intercepts were included for respondents and for UNESCO statements to account for repeated observations and statement-specific variability.

- **Outcome:** Continuous agreement score bounded between 0 and 1.
- **Stakeholder group:** "Knowledge Creator" serves as the reference category. Other groups include "Knowledge Mobiliser in Academia" and "Knowledge Mobiliser outside Academia".
- **Gender:** "Female" serves as the reference category.
- **Years of experience:** A continuous variable representing professional experience, included as a main effect and in interactions with stakeholder groups and gender to assess whether experience-related effects differ across groups.
- **Familiarity with open science:** Included as a fixed-effect predictor to assess its association with agreement with UNESCO open science statements.
- **Coding**: Categorical predictors were coded using treatment coding, such that all coefficients are interpreted relative to the reference levels.
- **Priors**: Weakly informative priors were applied: normal (0, 2.5) for fixed effects, and Student-t (3, 0, 1) priors for the standard deviations of the random intercepts.
- **Estimates**: For each fixed effect, the table reports posterior mean estimates, standard errors, and 95% credible intervals. Coefficients are reported on the logit scale of the mean of the Beta-distributed outcome. Positive estimates indicate higher predicted agreement relative to the reference condition. Interaction terms indicate whether the effect of years of experience varies by stakeholder group or gender.
- **Missing data**: The model accounts for missing data using multiple imputation, and results reflect the posterior distribution after combining information across imputations.

Model 5 - Question 4

Term	Estimate	Est.Error	l-95% CI	u-95% CI
**Years of Experience**	-0.031	0.011	-0.052	-0.010
**Knowledge Creator**	1.356	0.219	0.931	1.792
**Knowledge Mobiliser in Academia**	1.303	0.261	0.800	1.815
**Knowledge Mobiliser outside Academia**	1.764	0.247	1.283	2.253
**Familiarity OS**	0.756	0.220	0.325	1.181
**Gender (Male)**	-0.140	0.101	-0.337	0.055
**Years of experience:Knowledge Mobiliser in Academia**	0.028	0.021	-0.013	0.068
**Years of Experience:Knowledge Mobiliser outside Academia**	0.004	0.022	-0.039	0.047
**Years of Experience:Gender (Male)**	0.004	0.015	-0.025	0.032

Table S1.5. Posterior Summaries from a Bayesian Beta Regression Model for Agreement with UNESCO Open Science Statements

Model 5: Posterior Credible Intervals

Posterior samples were extracted for each model coefficient, and 95%, 80%, and 50% credible intervals (CI) were computed to visualise uncertainty in the parameter estimates.

Posterior estimates were grouped into three categories for plotting (see Figure S1.5):
A) **Intercepts**. Representing baseline agreement for the reference categories and random-effect structure;
B) **Continuous variable.** Years of experience, including main effects and interactions;
C) **Categorical variables**. Including stakeholder group, gender, familiarity with open science, and interaction terms.

For each parameter, posterior medians are shown as points, with horizontal bars representing the 50%, 80%, and 95% credible intervals. A vertical dashed line at $\beta = 0$ indicates the null (no-effect) value on the model's linear predictor scale, facilitating visual assessment of the direction and strength of effects across parameters.

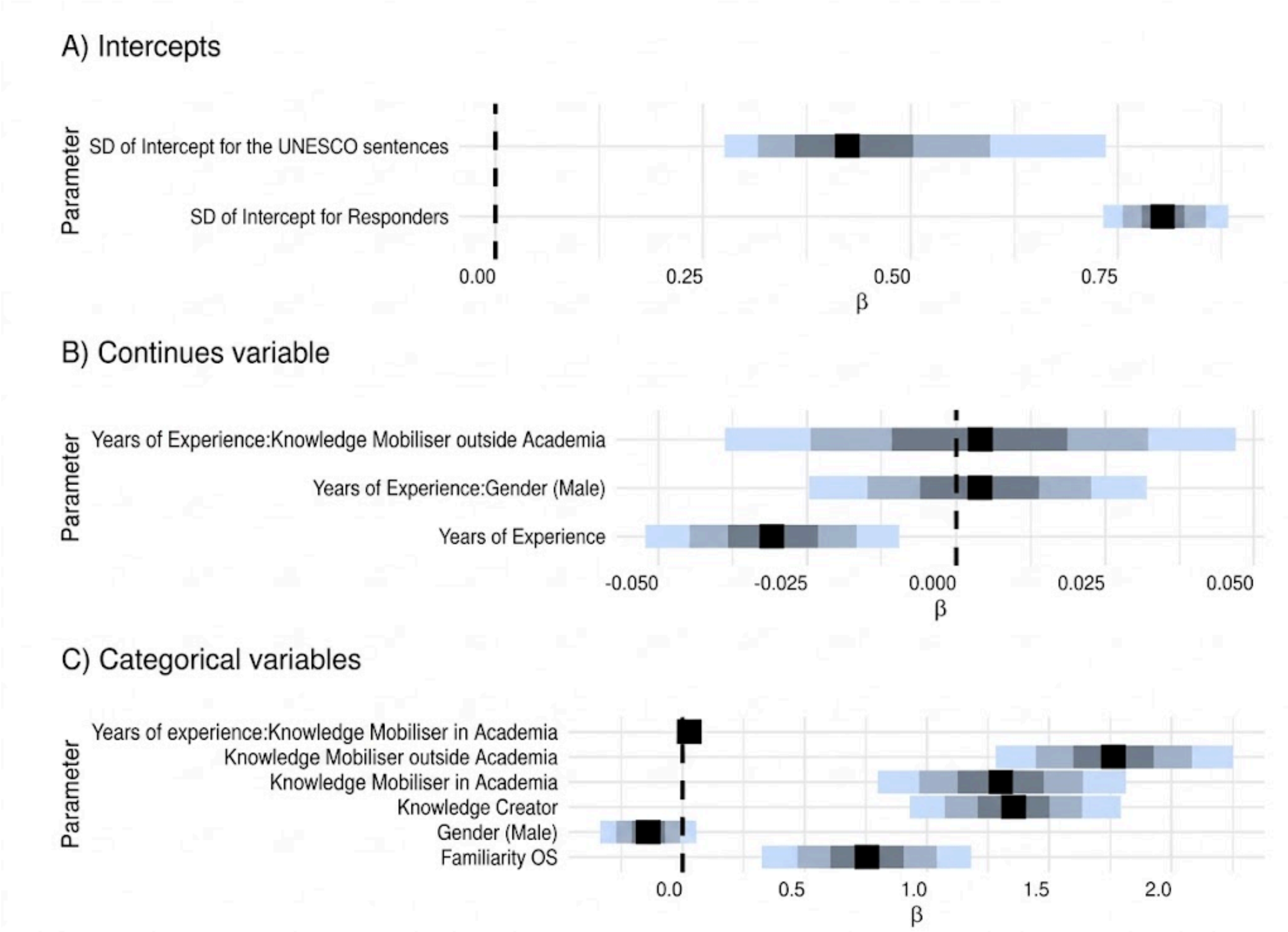

Figure S1.5. Posterior Credible Intervals for Model 5 (UNESCO Agreement)
